# Supplementary material for: Modified t-butyl in tetradentate platinum (II) complexes enables exceptional lifetime for blue-phosphorescent organic light-emitting diodes
Source: Nat Commun. 2024 Apr 6;15:2977. doi: 10.1038/s41467-024-47307-3 (PMC10998867; doi:10.1038/s41467-024-47307-3)
Supplement: Supplementary file 1 — Supplementary Information [file 41467_2024_47307_MOESM1_ESM.pdf]

Supplementary Information

Modified *t*-butyl in tetradentate platinum(II) complexes enables exceptional lifetime for blue-phosphorescent organic light-emitting diodes.

‡Young Hun Jung[1], ‡Gyeong Seok Lee[2], Subramanian Muruganantham[1], Hye Rin Kim[1], Jun Hyeog Oh[1], Jung Ho Ham[1], Sagar B. Yadav[1], Ji Hyun Lee[2], Mi Young Chae[1], Yun-Hi Kim[2], Jang Hyuk Kwon[1]**

[1]Organic Optoelectronic Device Lab (OODL), Department of Information Display
Kyung Hee University, 26, Kyungheedae-ro, Dongdaemun-gu, Seoul 02447, Republic of Korea.

[2]Gyeongsang National University, Jinju 52828, Republic of Korea.

**Corresponding Authors*: Yun-Hi Kim (ykim@gnu.ac.kr), Jang Hyuk Kwon (jhkwon@khu.ac.kr)

[a] These authors contributed equally to this work.

Table of contents

1. Supplementary Methods

1.1 Measurements

1.2 Synthetic procedure and Characterization

2. Supplementary Figures

Supplementary Figure 1. Synthetic route of Pt complexes (PtON-TBBI, PtON-tb-DTB(Pt-1), PtON-tb-TTB(Pt-2)).

Supplementary Figure 2. ^{1}H-NMR spectrum of PtON-TBBI

Supplementary Figure 3. ^{13}C-NMR spectrum of PtON-TBBI

Supplementary Figure 4. Q-Tof mass data of PtON-TBBI

Supplementary Figure 5. ^{1}H-NMR spectrum of Intermediate material (**3**)

Supplementary Figure 6. ^{13}C-NMR spectrum of Intermediate material **(3)**

Supplementary Figure 7. QTof mass data of Intermediate material **(3)**

Supplementary Figure 8. ^{1}H-NMR spectrum of Intermediate material **(4)**

Supplementary Figure 9. ^{13}C-NMR spectrum of Intermediate material **(4)**

Supplementary Figure 10. QTof mass data of Intermediate material **(4)**

Supplementary Figure 11. ^{1}H-NMR spectrum of PtON-tb-TTB

Supplementary Figure 12. ^{13}C-NMR spectrum of PtON-tb-TTB

Supplementary Figure 13. QTof mass data of PtON-tb-TTB

Supplementary Figure 14. ^{1}H-NMR spectrum of Intermediate material **(5)**

Supplementary Figure 15. ^{13}C-NMR spectrum of Intermediate material **(5)**

Supplementary Figure 16. QTof mass data of Intermediate material **(5)**

Supplementary Figure 17. ^{1}H-NMR spectrum of PtON-tb-TTB

Supplementary Figure 18. ^{13}C-NMR spectrum of PtON-tb-TTB

Supplementary Figure 19. QTof mass data of PtON-tb-TTB

Supplementary Figure 20. TGA thermograms of PtON-TBBI, PtON-tb-DTB, and PtON-tb-TTB

Supplementary Figure 21. DSC thermograms of platinum complexes (DSC data for PtON-TBBI, PtON-tb-DTB, Pt-tb-TTB are shown in the top figure in order).

Supplementary Figure 22. Cyclic-Voltammetry measurements of PtON-TBBI, PtON-tb-DTB, Pt-tb-TTB.

Supplementary Figure 23. TRPL measurements of PtON-tb-DTB and PtON-tb-TTB according to doping concentration on PMMA matrix.

Supplementary Figure 24. Expected and Measured PLQY values of PtON-TBBI, PtON-tb-DTB, and PtON-tb-TTB according to doping concentration in PMMA matrix.

Supplementary Figure 25. Device energy diagram and molecular structures.

Supplementary Figure 26. Normalized Im$|Z|$ – Frequency measurements of HOD and EOD

Supplementary Figure 27. UV-stability test of PtON-TBBI, PtON-tb-DTB, and PtON-tb-TTB (UV-Lamp: 360nm)

Supplementary Figure 28. Luminescence (L) vs Current Density (J) curve

Supplementary Figure 29. DFT simulation of PtON-tb-MTB

3. Supplementary Tables

Supplementary Table 1. Measured exciton lifetime according to doping ratio.

Supplementary Table 2. Calculated FRET and DET rate according to intermolecular distance.

4. Supplementary Discussion

4.1 Forster Resonance Energy Transfer (FRET) Rate

4.2 Dexter Energy Transfer (DET) Rate

4.3 Roll-off Analysis.

5. Supplementary References

1. Supplementary Methods

1.1 Measurements

Proton nuclear magnetic resonance (^{1}H NMR) and carbon nuclear magnetic resonance (^{13}C NMR) spectra were acquired on a Bruker DRX 300 MHz spectrometer. High-resolution mass spectra (HRMS) were obtained using quadrupole time of flight (QTof) methods with a Xevo G2-XS Tof. Thermogravimetric analysis (TGA) was performed using a TA 2050 TGA thermogravimetric analyzer under a nitrogen atmosphere. The sample was heated at a rate of 10 °C/min. Differential scanning calorimetry (DSC) was performed using a TA Instruments 2100 DSC under a nitrogen atmosphere. The sample was heated at a rate of 10 ℃/min from 0 ℃ to 300 ℃. The absorption spectrum was measured using a PerkinElmer LAMBDA-900 UV/Vis/IR spectrophotometer. The room temperature (300 K) photoluminescence (PL) spectra were measured using an LS-50B luminescence spectrophotometer. Cyclic voltammetry was measured on a ZIVE SP1 Wonatech at room temperature using a 0.1 M solution of tetrabutylammonium perchlorate (Bu_4NClO_4) in N,N-dimethylformamide at a scanning rate of 20 mV/s. A Pt wire was used as the counter electrode and an Ag/Ag+ electrode was used as the reference electrode. The final products were purified by vacuum sublimation prior to measurements and device fabrication.

1.2 Synthetic procedure and characterization

Synthesis of PtON-TBBI (Pt-ref)

In a dried 250 ml three-neck round bottom flask was added intermediate (2) (5.0 g, 5.91 mmol), Pt(COD)Cl_2 (2.32 g, 6.21 mmol), sodium acetate (1.45 g, 17.73 mmol), and benzonitrile (300 mL). The flask was backfilled with nitrogen. The reaction mixture was stirred at 180˚C under nitrogen atmosphere for 24 h. After cooling to room temperature, the solvent was removed under reduced pressure to obtain a crude product. The crude product was purified by column chromatography on silica gel (methylene chloride: acetone = 1:10) to afford the desired product as yellow solid (3.2 g, 62 %). ^{1}H-NMR (300 MHz, CD_2Cl_2): δ (ppm) = 8.78 – 8.75 (m, 1H), 8.27 (d, *J* = 8.4 Hz, 1H), 8.10 – 8.07 (m, 1H), 7.88 (d, *J* = 8.4 Hz, 1H), 7.82 (d, *J* = 1.8 Hz, 1H), 7.78 – 7.75 (m, 1H), 7.62 – 7.53 (m, 4H), 7.48 – 7.35 (m, 6H), 7.29 (t, *J* = 8.1 Hz, 1H), 7.14 – 7.06 (m, 1H), 6.00 (dd, *J* = 6.3 Hz, 1.8 Hz, 1H) 1.32 (s, 18H), 1.09 (s, 9H). ^{13}C-NMR (300 MHz, CD_2Cl_2): δ (ppm) = 191.2, 162.0, 155.3, 153.9, 152.0, 147.9, 138.7, 136.4, 136.2, 132.1, 128.8, 124.7, 124.3, 123.7, 123.2, 122.6, 122.2, 119.8, 116.1, 115.4, 115.3, 114.1, 113.5, 112.2, 112.0, 111.9, 111.8, 107.5, 35.0, 34.8, 31.2, 29.6. HRMS (QTof, m/z): $[M+H]^+$ = 890.3398 calcd for $C_{48}H_{46}N_4OPt$, found 890.3396.

Synthesis of 2-(3-(1*H*-benzo[*d*]imidazol-1-yl)-5-(*tert*-butyl)phenoxy)-9-(4-(*tert*-butyl)pyridin-2-yl)-9*H*-carbazole, Intermediate material (3)

To a three-neck round bottom flask were added 1-(3-bromo-5-(tert-butyl)phenyl)-1H-benzo[d]imidazole (6 g, 18.22 mmol), 9-(4-(tert-butyl)pyridin-2-yl)-9H-carbazol-2-ol (6.34 g, 20.04 mmol), copper(I) iodide (1.04 g, 5.46 mmol), tripotassium phosphate (15.46 g, 72.88 mmol), picolinic acid (3.58 g, 29.15 mmol), and DMSO (130 mL). The reaction mixture was stirred at 110˚C for 24 h. After cooling to room temperature, the reaction mixture was diluted with water and extracted with ethyl acetate. The organic layer was dried over anhydrous $MgSO_4$. After the solvent was evaporated and the residue was purified by column chromatography on silica gel (ethyl acetate: hexane = 1:5) to afford the desired product as pale-yellow solid (7.6 g, 73%). ^{1}H-NMR (300 MHz, $CDCl_3$): δ (ppm) = 8.61 (d, *J* = 5.4 Hz, 1H), 8.29 – 8.09 (m, 3H), 7.90 – 7.89 (m, 1H), 7.76 (d, *J* = 5.1 Hz, 1H), 7.64 – 7.57 (m, 3H), 7.48 – 7.42 (td,

J = 7.2Hz , 1.2 Hz, 1H), 7.37 – 7.28 (m, 4H), 7.25 (t, *J* = 1.8 Hz, 2H), 7.12 (dd, *J* = 8.4 Hz, 2.1 Hz, 1H) 7.01 (t, *J* = 1.8 Hz, 1H), 1.38 (s, 9H), 1.37 (s, 9H). ^{13}C-NMR (300 MHz, $CDCl_3$): δ (ppm) = 163.1, 159.2, 155.4, 155.0, 149.4, 140.7, 140.1, 125.8, 123.9, 123.9, 122.9, 121.2, 121.1, 119.9, 118.8, 116.2, 115.3, 115.2, 113.0, 110.8, 110.6, 102.6, 35.2, 35.1, 31.2, 30.5. HRMS (QTof, m/z): $[M+H]^+$ = 565.2967 calcd for $C_{38}H_{36}N_4O$, found 565.2971.

Synthesis of 1*H*-Benzimidazolium, 3-[3-(1,1-dimethylethyl)-5-[[9-[4-(1,1-dimethylethyl)-2-pyridinyl]-9*H*-carbazol-2-yl]oxy]phenyl]-1-[3-(1,1-dimethylethyl)phenyl]-, 1,1,1-trifluoromethanesulfonate, Intermediate material (4)

To a three-neck round bottom flask were added compound (3) (7.5 g, 13.28 mmol), (3-tert-butylphenyl)(mesityl)iodonium trifluoromethane sulfonate (10.48 g, 19.92 mmol), copper(II) acetate (0.23 g, 1.32 mmol), and DMF (65 mL). The reaction mixture was stirred at 130˚C for 12 h. After cooling to room temperature, the reaction mixture was diluted with water and extracted with ethyl acetate. The organic layer was dried over anhydrous $MgSO_4$. After the solvent was evaporated, the crude product was purified by column chromatography on silica gel (methylene chloride: acetone =1:10) to afford the desired product as pale yellow (6.4 g, 53 %). ^{1}H-NMR (300 MHz, $CDCl_3$): δ (ppm) = 10.08 (s, 1H), 8.61 (d, *J* = 6.0 Hz, 1H), 8.15 (t, *J* = 9.0 Hz, 2H), 7.79 – 7.62 (m, 12H), 7.48 – 7.28 (m, 5H), 7.16 (d, *J* = 9.0 Hz, 1H), 7.05 (s, 1H), 1.41 (m, 27H). ^{13}C-NMR (300 MHz, $CDCl_3$): δ (ppm) = 163.4, 162.5, 159.5, 157.1, 154.4, 151.4, 149.5, 140.9, 140.8, 140.1, 133.0, 132.2, 131.8, 131.7, 130.4, 128.3, 128.2, 128.0, 125.9, 123.8, 122.6, 122.2, 121.4, 121.1, 120.1, 119.1, 118.3, 117.9, 117.1, 116.3, 114.0, 113.8, 113.4, 110.6, 110.5, 103.3, 35.5, 35.2, 35.1, 31.1, 31.0, 30.5. HRMS (QTof, m/z): 697.3901 calcd for $[C_{48}H_{49}N_4O]^+$, found 697.3903.

Synthesis of PtON-tb-DTB (Pt-1)

To a three-neck round bottom flask were added intermediate material (4) (6 g, 7.08 mmol), $Pt(COD)Cl_2$ (2.64 g, 7.08 mmol), sodium acetate (1.74 g, 21.24 mmol), and benzonitrile (360 mL). The flask was backfilled with nitrogen. the reaction mixture was stirred at 180˚C under nitrogen atmosphere for 24 h. After cooling to room temperature, the solvent was removed under reduced pressure to obtain a crude product. The crude product was purified by column chromatography on silica gel (methylene chloride: acetone = 1:10) to afford the desired product as yellow solid (3.1 g, 49 %). ^{1}H-NMR (300 MHz, CD_2Cl_2): δ (ppm) = 8.76 – 8.74 (m, 1H), 8.08 – 8.04 (m, 2H), 7.86 (d, *J* = 8.1 Hz, 1H), 7.77 – 7.62 (m, 4H), 7.55 – 7.49 (m, 1H), 7.44 – 7.28 (m, 9H), 6.07 – 6.05 (dd, *J* = 6.3 Hz, 1.5 Hz, 1H), 1.60 (s, 9H), 1.13 (s, 9H), 1.00 (s, 9H). ^{13}C-NMR (300 MHz, $CDCl_3$): δ (ppm) = 161.3, 154.4, 152.8, 152.4, 150.0, 148.3, 147.4, 143.8, 138.6, 136.5, 136.0, 131.8, 129.0, 128.7, 125.4, 124.7, 124.0, 123.3, 122.8, 122.0, 119.9, 116.4, 116.3, 115.6, 113.9, 112.5, 112.2, 111.7, 111.1, 110.9, 107.4, 105.6, 34.9, 34.5, 31.7, 31.1, 29.5. HRMS (QTof, m/z): $[M+H]^+$ = 890.3398 calcd for $C_{52}H_{54}N_4OPt$, found 890.3392.

Synthesis of 1*H*-Benzimidazolium, 1-[3,5-bis(1,1-dimethylethyl)phenyl]-3-[3-[[9-[4-(1,1-dimethylethyl)-2-pyridinyl]-9*H*-carbazol-2-yl]oxy]-5-(1,1-dimethylethyl)phenyl], 1,1,1-trifluoromethanesulfonate, Intermediate material (5)

The synthetic procedure for Intermediate material (5) was identical to that of Intermediate material (4), except that (3,5-di-tert-butylphenyl)(mesityl)iodonium trifluoromethane sulfonate was used instead of (3-tert-butylphenyl)(mesityl)iodonium trifluoromethane sulfonate. (Yield: 51 %) ^{1}H-NMR (300 MHz, $CDCl_3$): δ (ppm) = 10.03 (s, 1H), 8.62 (d, *J* = 6.0 Hz, 1H), 8.15 (t, *J* = 9.0 Hz, 2H), 7.78 – 7.53 (m, 12H), 7.48 – 7.42 (m, 6H), 7.37 – 7.28 (m, 4H), 7.17 (d, *J* = 9.0 Hz, 1H), 7.03 (s, 1H), 1.43-1.37 (m, 36H). ^{13}C-NMR (300 MHz, $CDCl_3$): δ (ppm) = 163.3, 159.5, 157.0, 154.4, 154.0, 151.4, 149.5, 140.9, 140.8, 140.1, 133.0, 132.0, 131.7, 128.0, 127.9, 125.9, 125.2, 123.8, 121.4, 121.1, 121.0, 120.0, 119.6, 119.1, 117.9, 117.3, 116.3, 113.9, 113.5, 110.7, 110.6, 103.3, 35.6, 35.3, 35.1, 31.2, 31.0, 30.5. HRMS (QTof, m/z): 753.4527 calcd for $[C_{52}H_{57}N_4O]^+$, found 753.4542.

Synthesis of PtON-tb-TTB (Pt-2)

The synthetic procedure for PtON-tb-TTB was identical to that of PtON-tb-DTB except using Intermediate material (5) instead of Intermediate material (4). (Yield: 52%) ^{1}H-NMR (300 MHz, CD_2Cl_2): δ (ppm) = 8.76 – 8.74 (m, 1H), 8.30 (d, 2H, *J* = 8.1 Hz), 8.11 – 8.08 (m, 1H), 7.97 (d, 4H, *J* = 1.8 Hz), 7.88 – 7.77 (m, 3H), 7.70 (d, 2H, *J* = 1.2 Hz) 7.55 – 7.50 (m, 2H), 7.46 – 7.38 (m, 5H), 7.37 – 7.15 (m, 1H), 6.25 (dd, 1H, *J* = 6.3 Hz, 2.1 Hz), 1.56 (s, 9H), 1.35 – 1.25 (m, 27H). ^{13}C-NMR (300 MHz, $CDCl_3$): δ (ppm) = 191.3, 161.8, 154.9, 154.5, 152.0, 148.5, 148.4, 143.9, 142.0, 138.7, 136.4, 136.4, 132.2, 129.2, 124.8, 123.4, 123.2, 122.5, 122.2, 120.0, 116.5, 115.6, 115.5, 114.8, 113.9, 112.7, 112.4, 111.9, 111.9, 111.0, 106.5, 105.6, 35.2, 35.0, 34.9, 31.6, 31.5, 30.0. HRMS (QTof, m/z): $[M+H]^+$ = 946.4018 calcd for $C_{52}H_{54}N_4OPt$, found 946.4024.

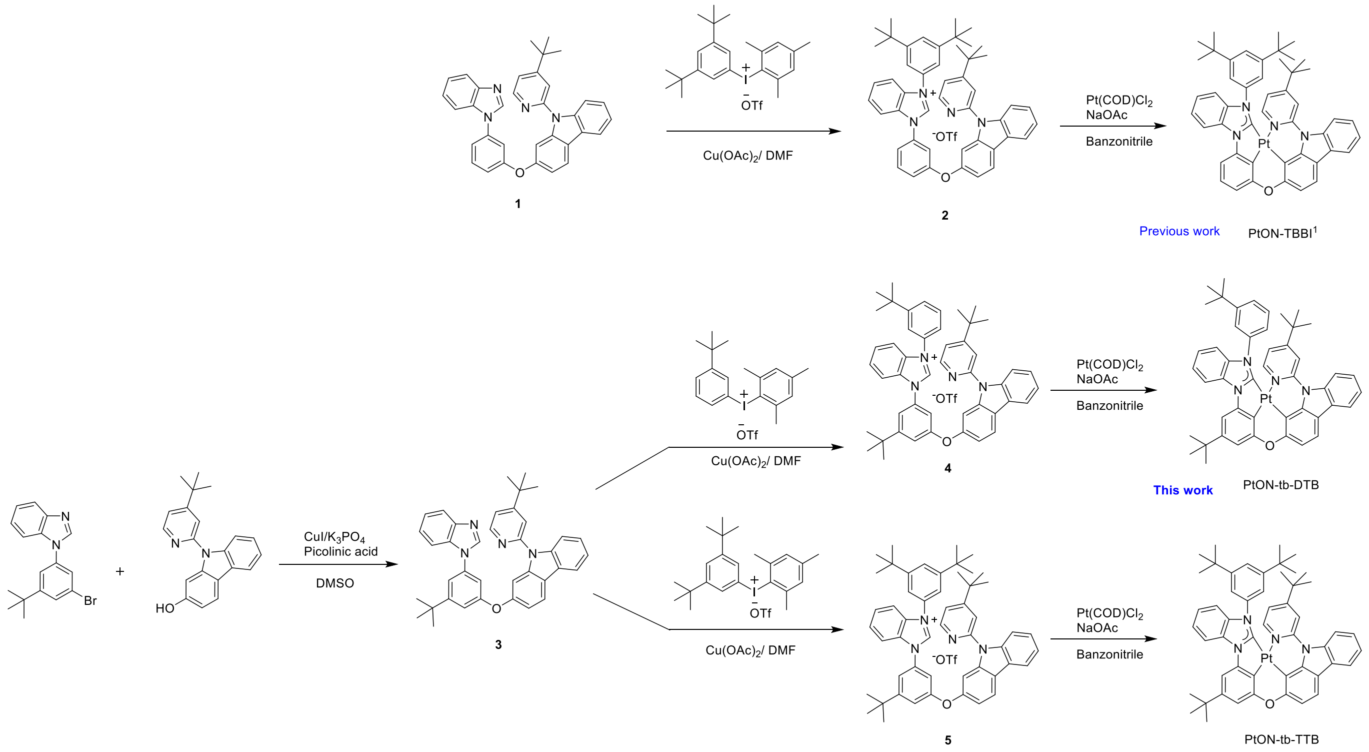

Supplementary Figure 1. Synthetic route of Pt(II) complexes (PtON-TBBI, PtON-tb-DTB & PtON-tb-TTB)[1].

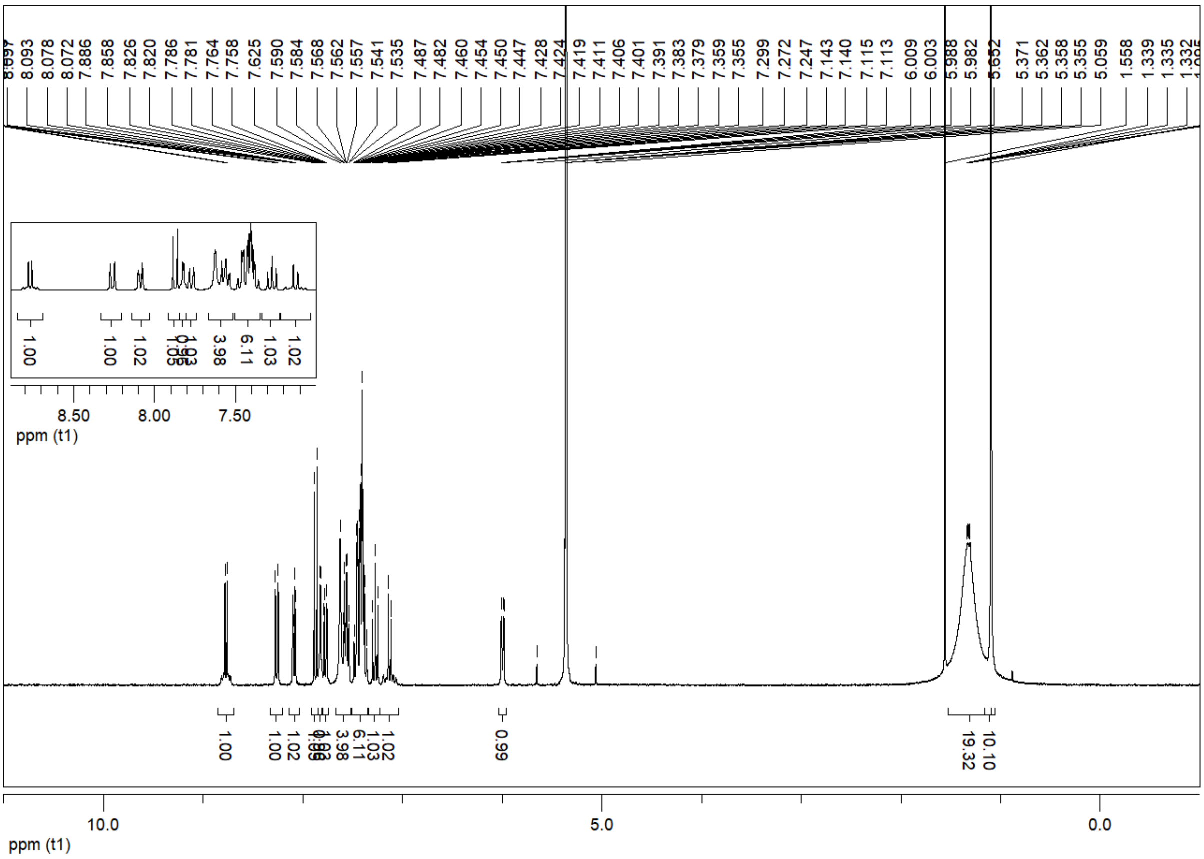

Supplementary Figure 2. ^{1}H-NMR spectrum of PtON-TBBI

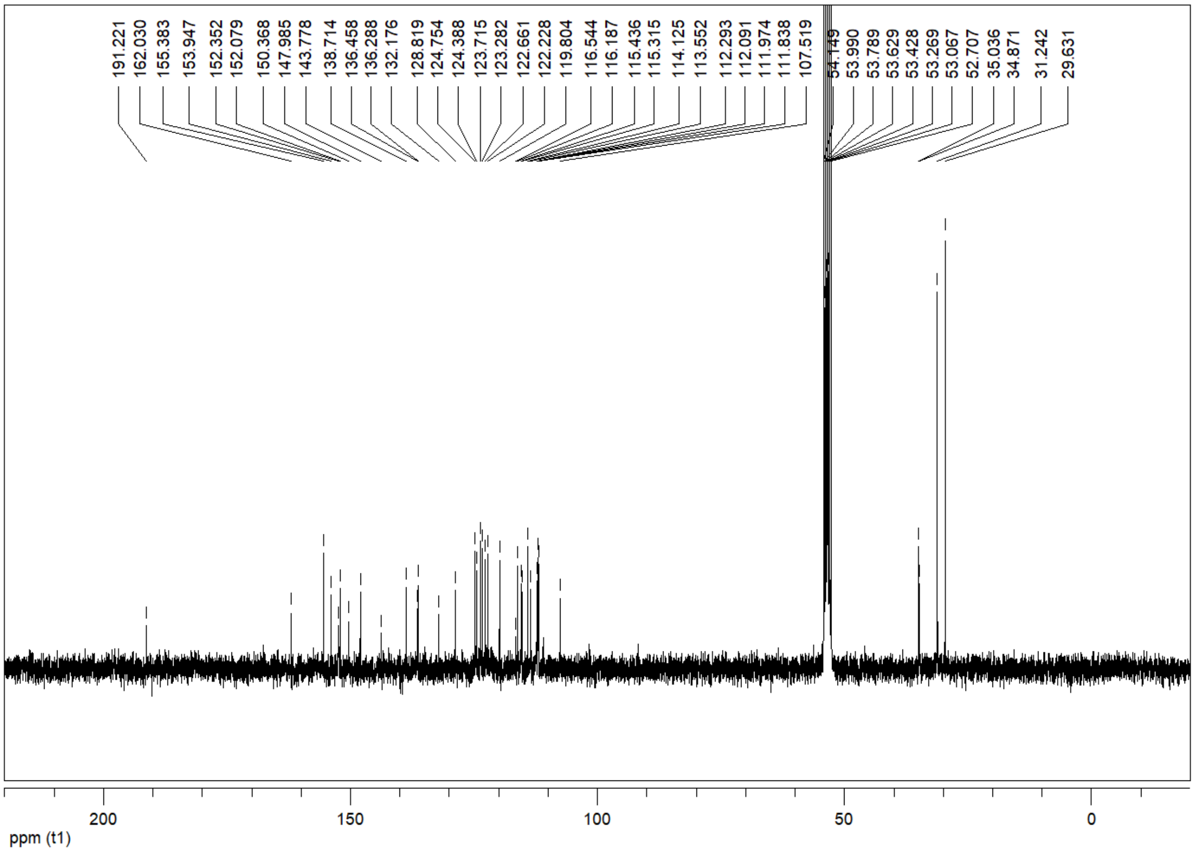

Supplementary Figure 3. ^{13}C-NMR spectrum of PtON-TBBI

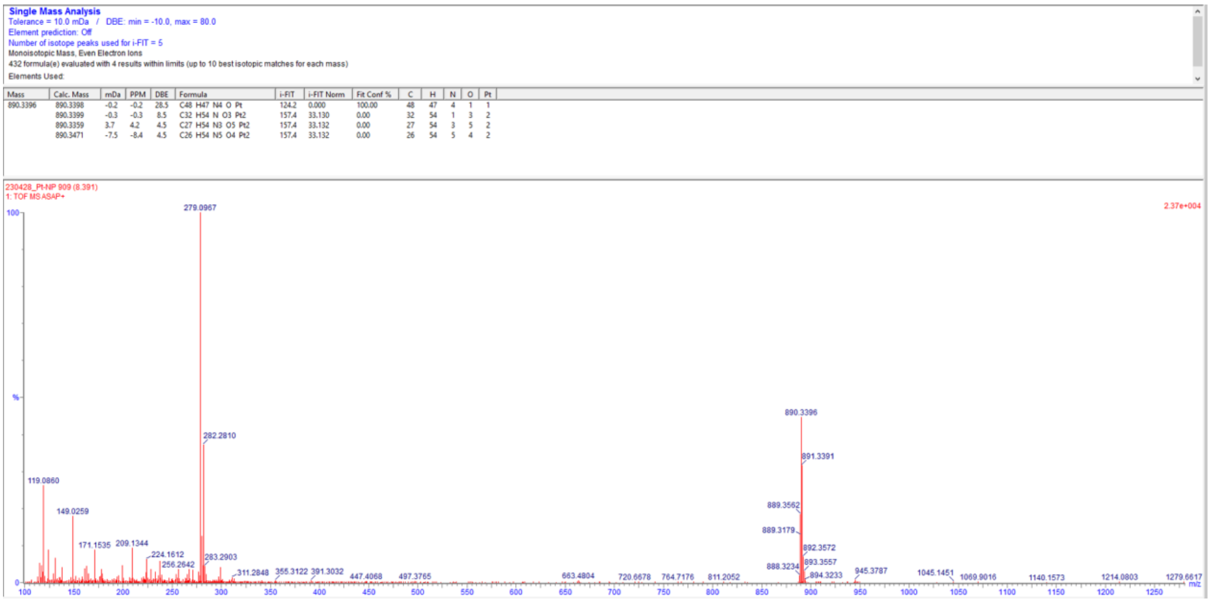

Supplementary Figure 4. QTof mass data of PtON-TBBI

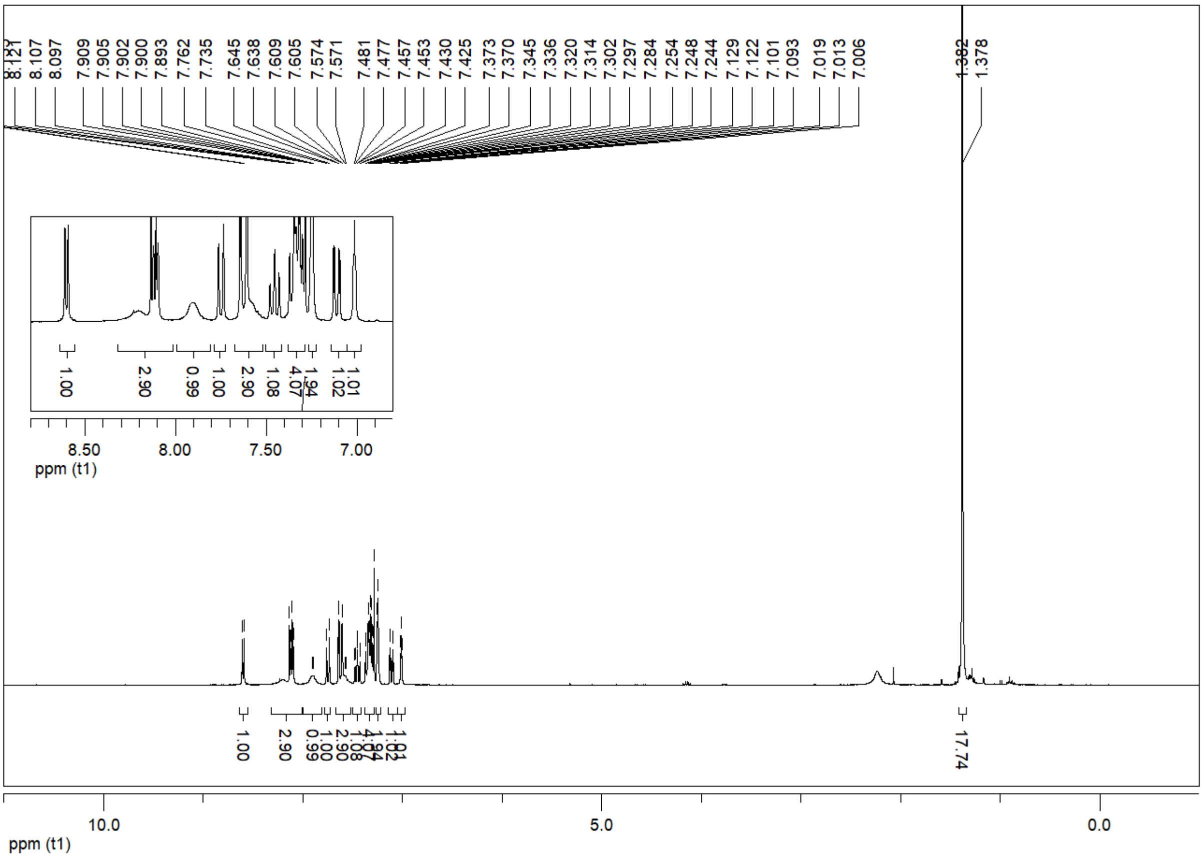

Supplementary Figure 5. ^{1}H-NMR spectrum of Intermediate material (3)

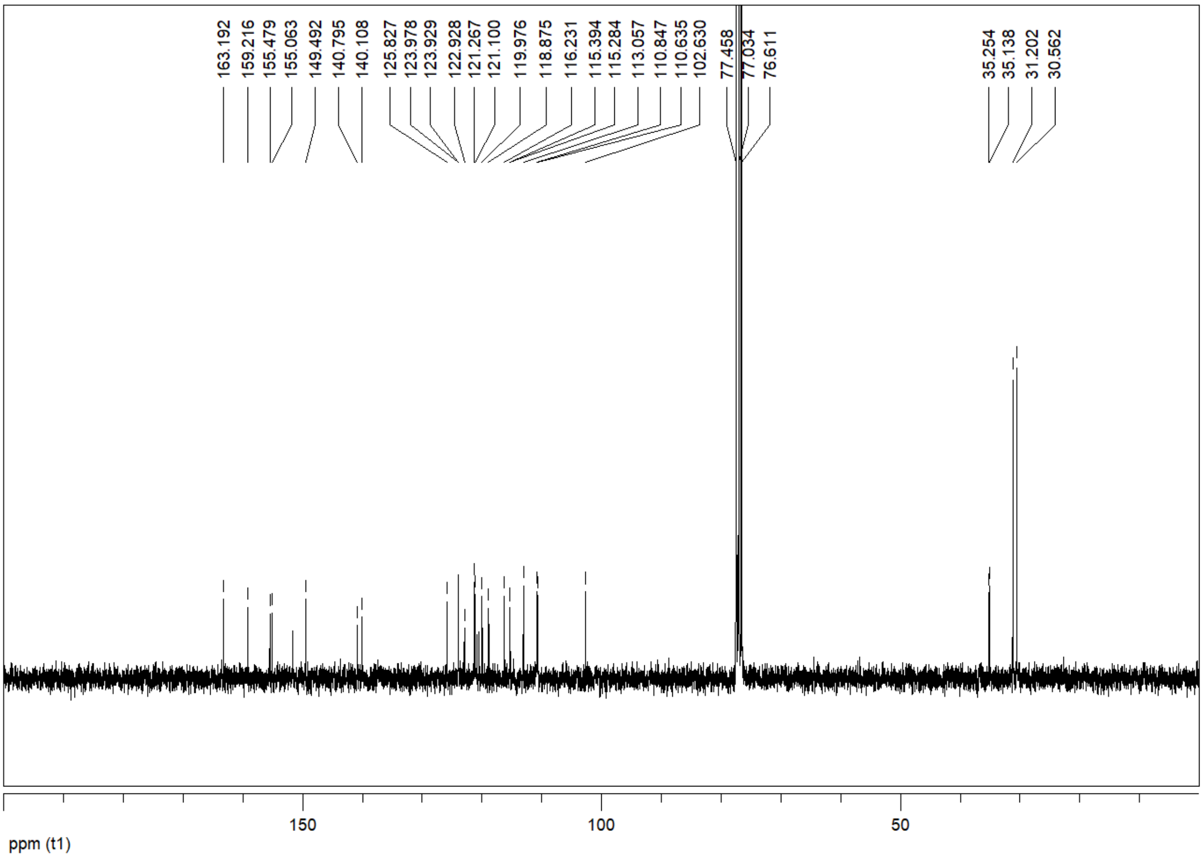

Supplementary Figure 6. ^{13}C-NMR spectrum of Intermediate material (3)

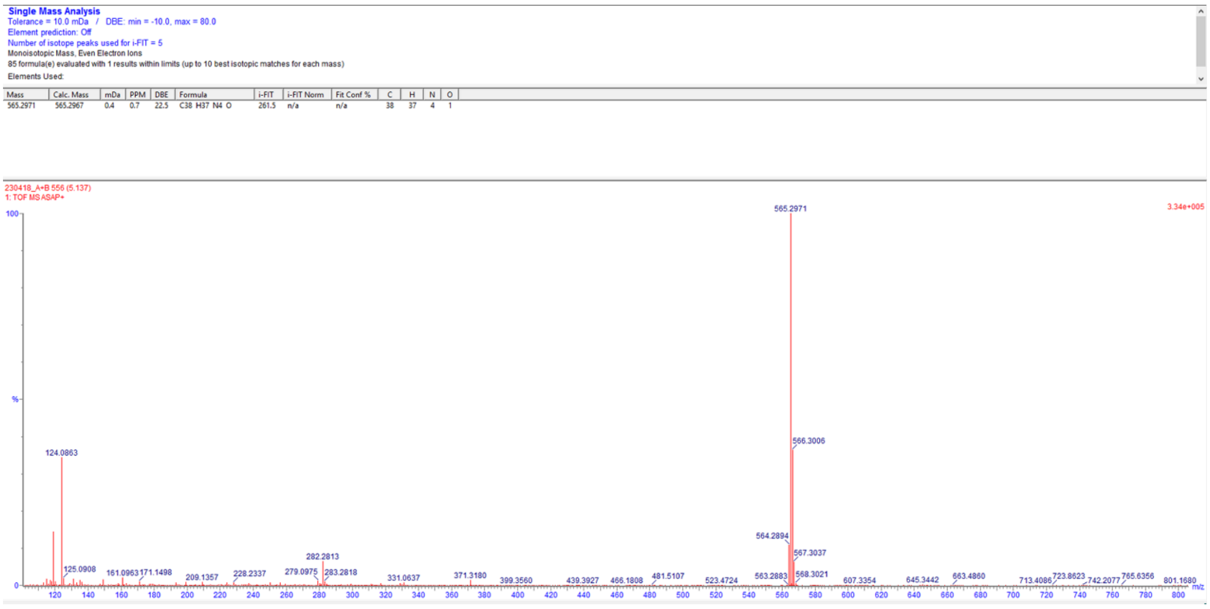

Supplementary Figure 7. QTof mass data of Intermediate material (3)

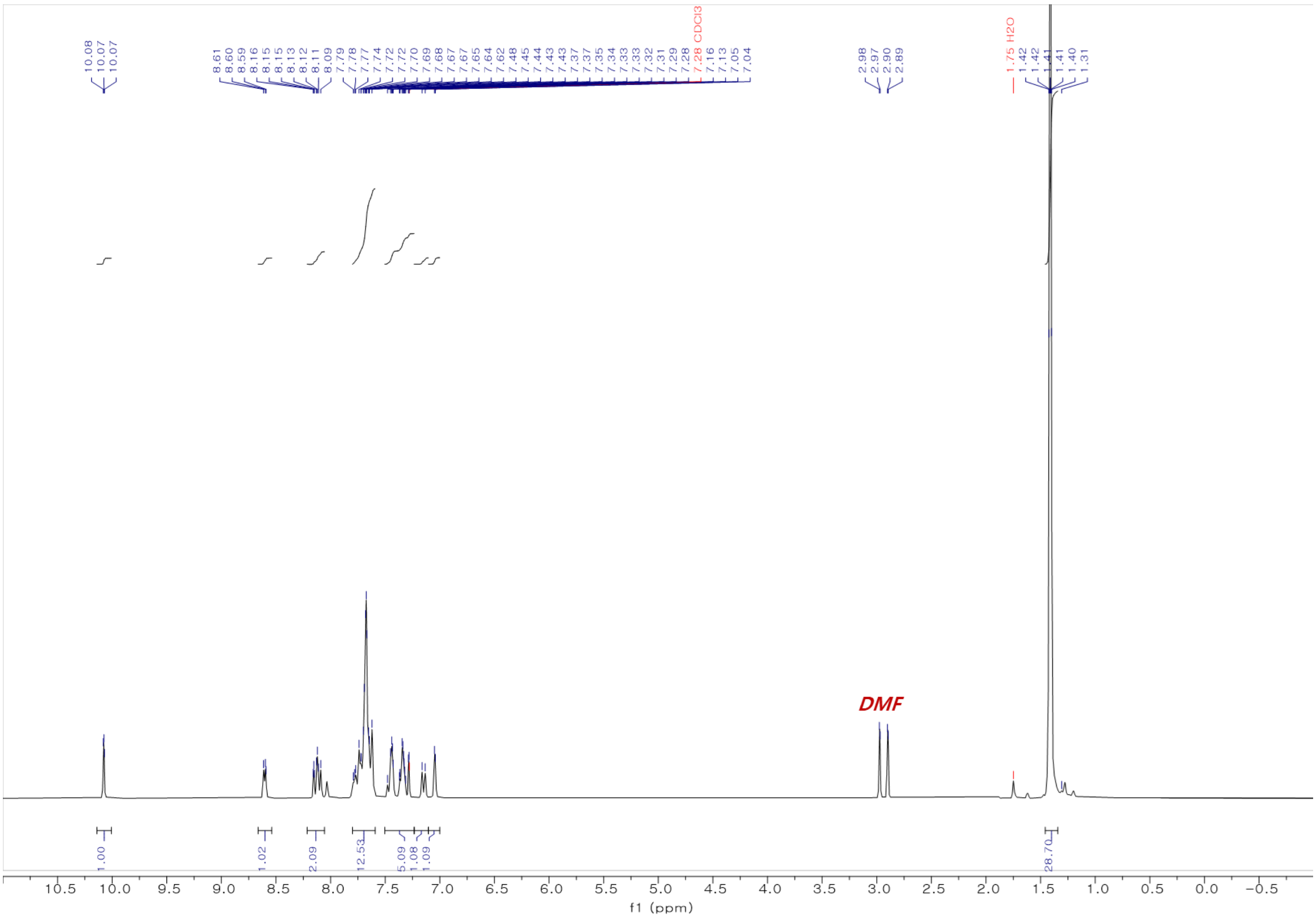

Supplementary Figure 8. ^{1}H-NMR spectrum of Intermediate material (4)

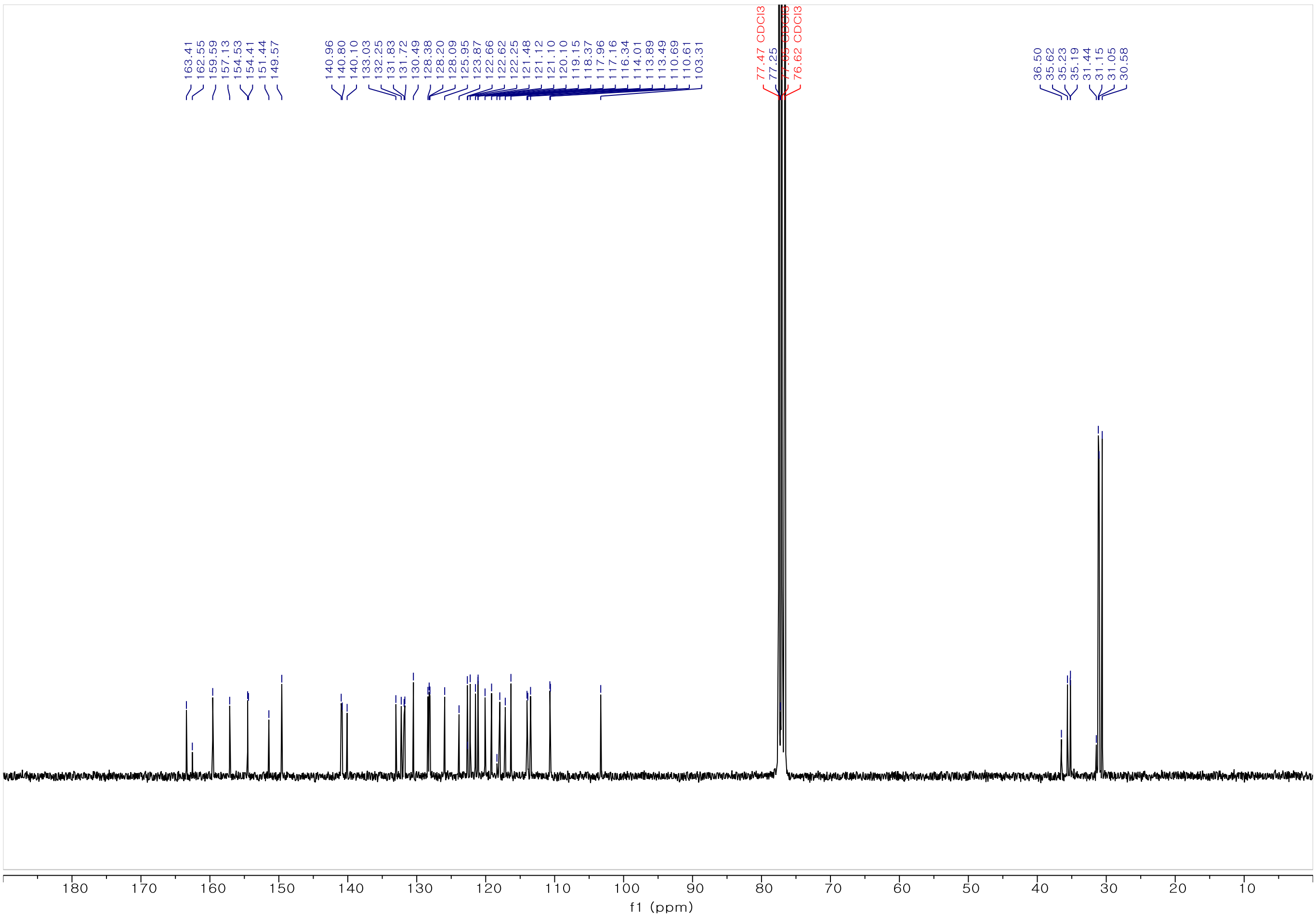

Supplementary Figure 9. ^{13}C-NMR spectrum of Intermediate material (4)

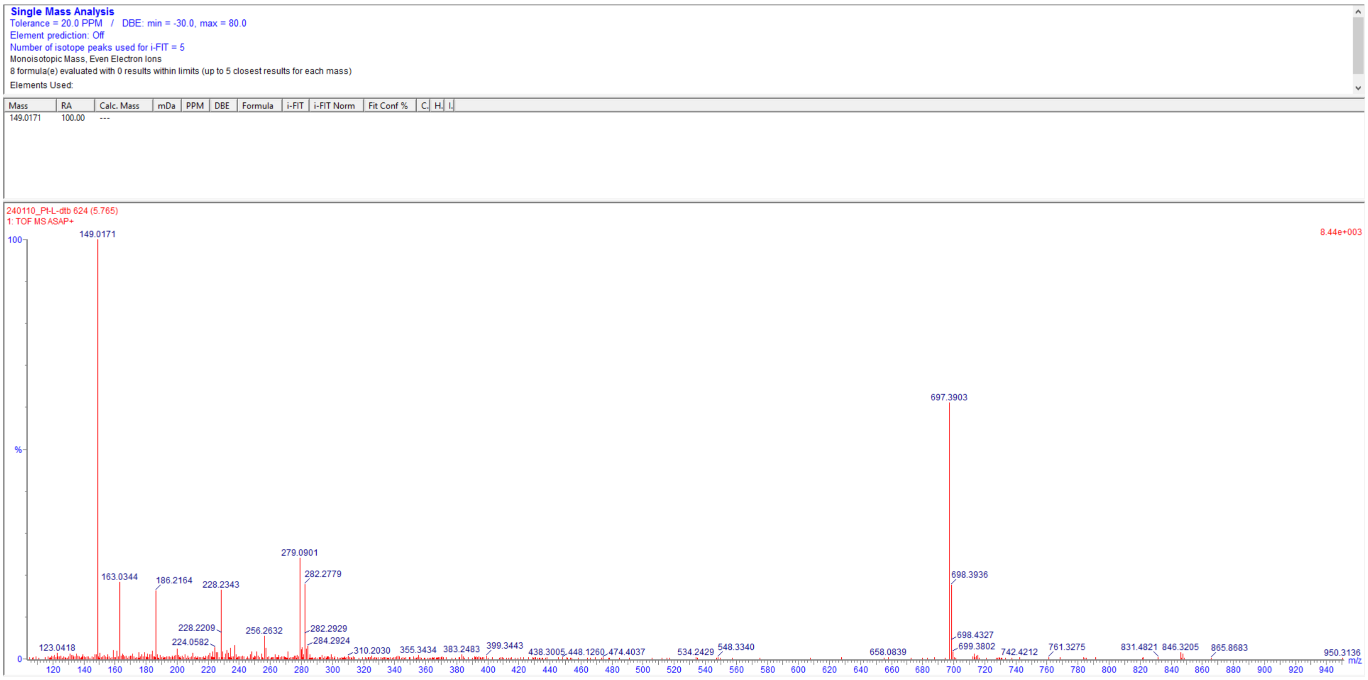

Supplementary Figure 10. QTof mass data of Intermediate material (4)

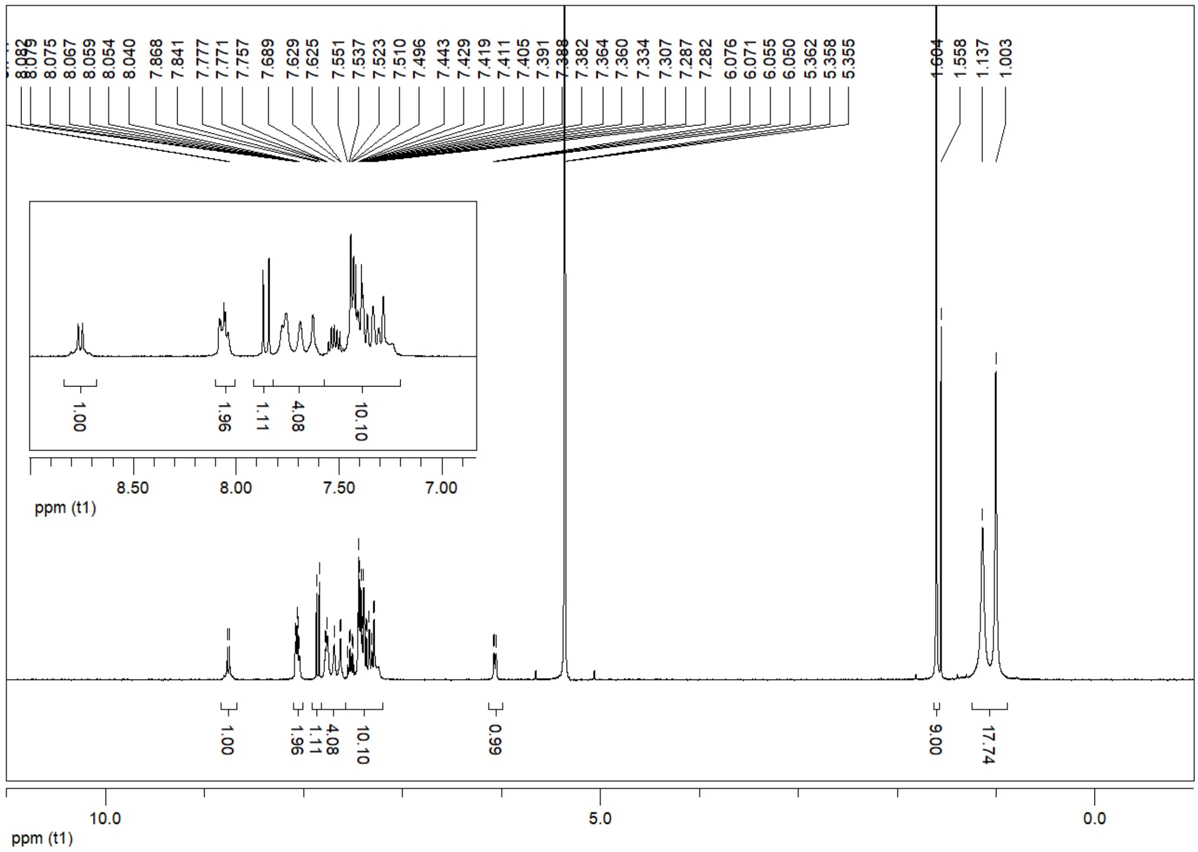

Supplementary Figure 11. ^{1}H-NMR spectrum of PtON-tb-DTB

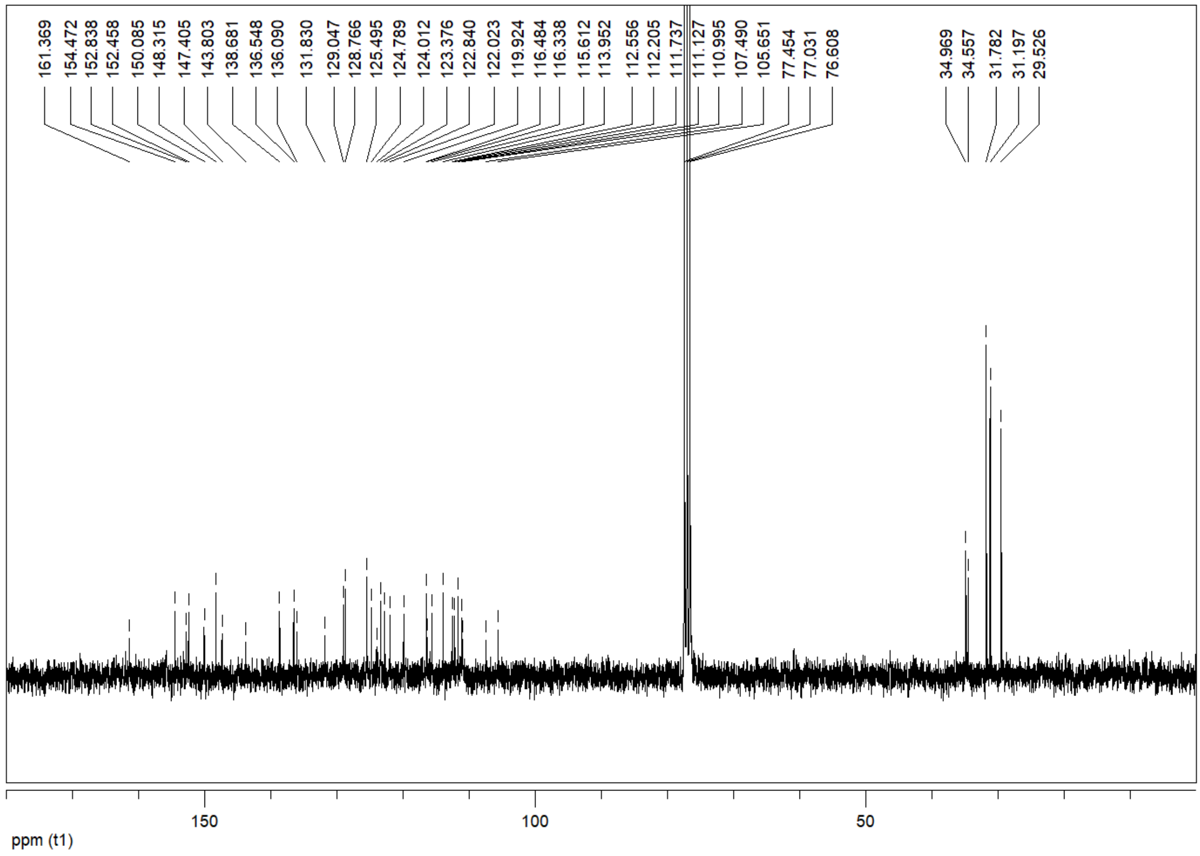

Supplementary Figure 12. ^{13}C-NMR spectrum of PtON-tb-DTB

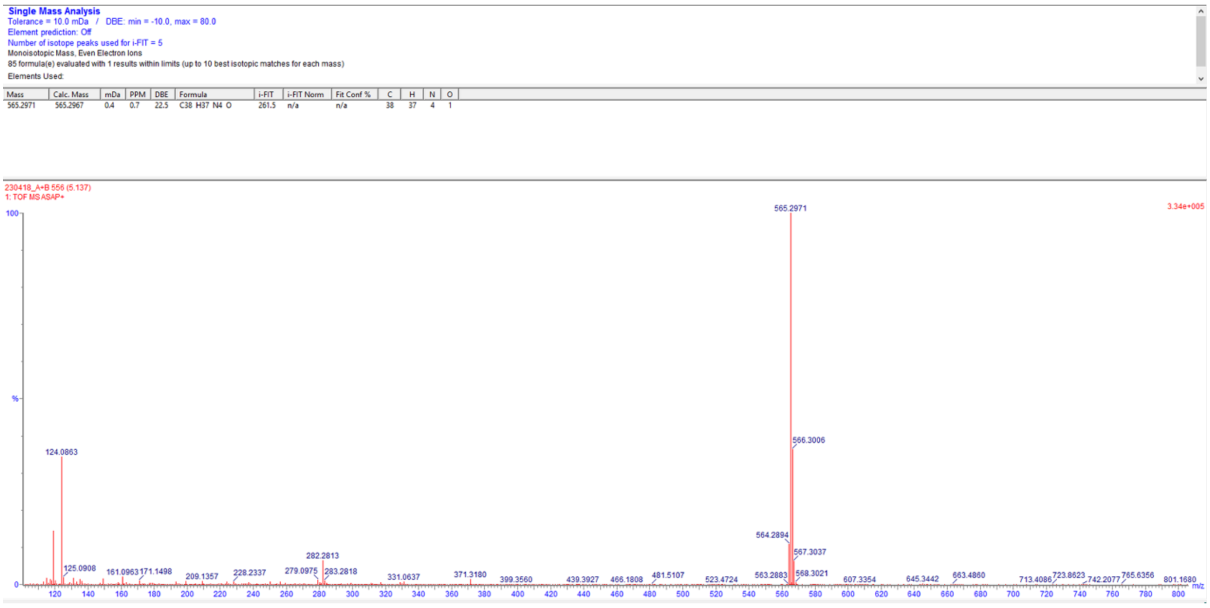

Supplementary Figure 13. QTof mass data of PtON-tb-DTB

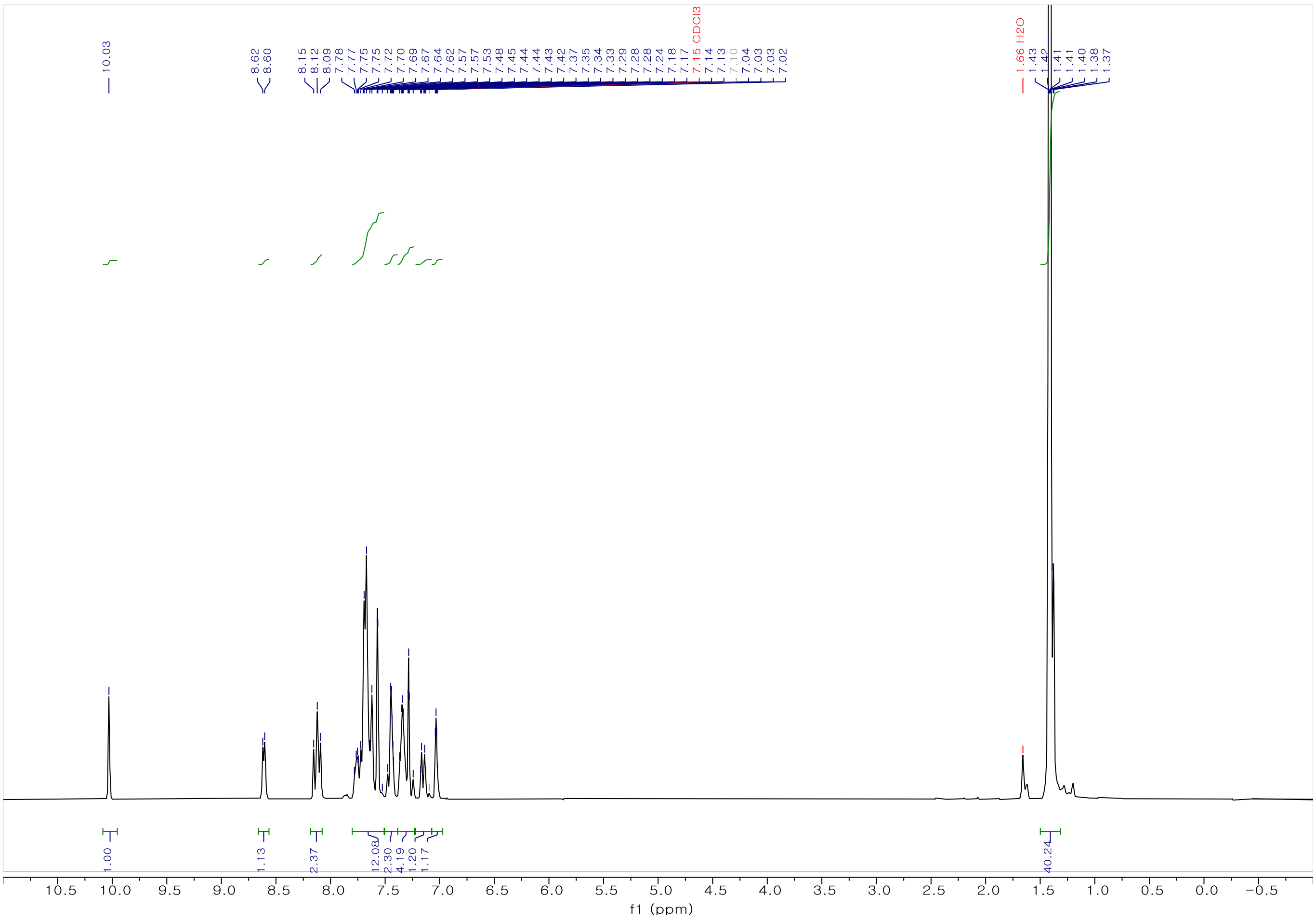

Supplementary Figure 14. ^{1}H-NMR spectrum of Intermediate material (5)

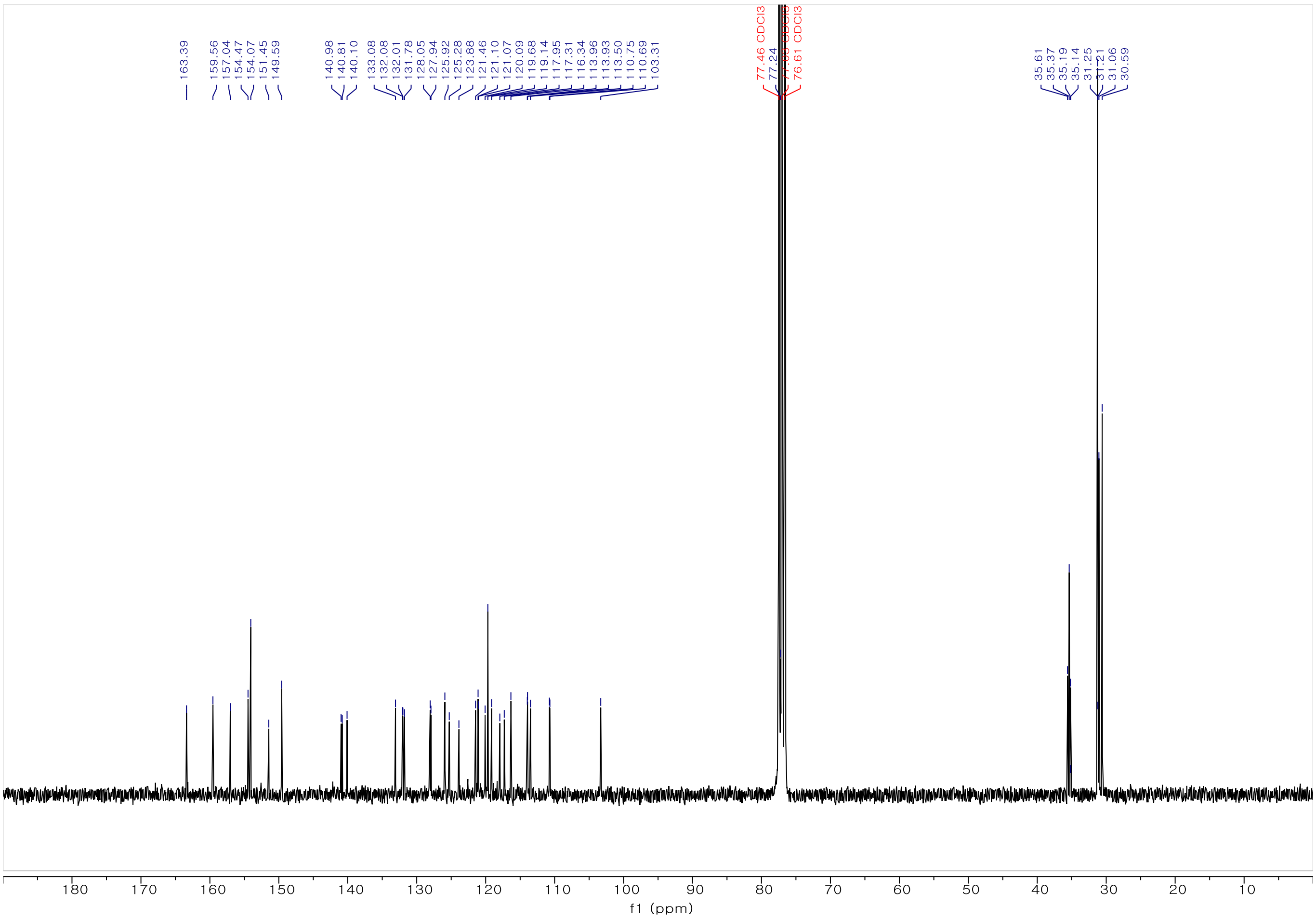

Supplementary Figure 15. ^{13}C-NMR spectrum of Intermediate material (5)

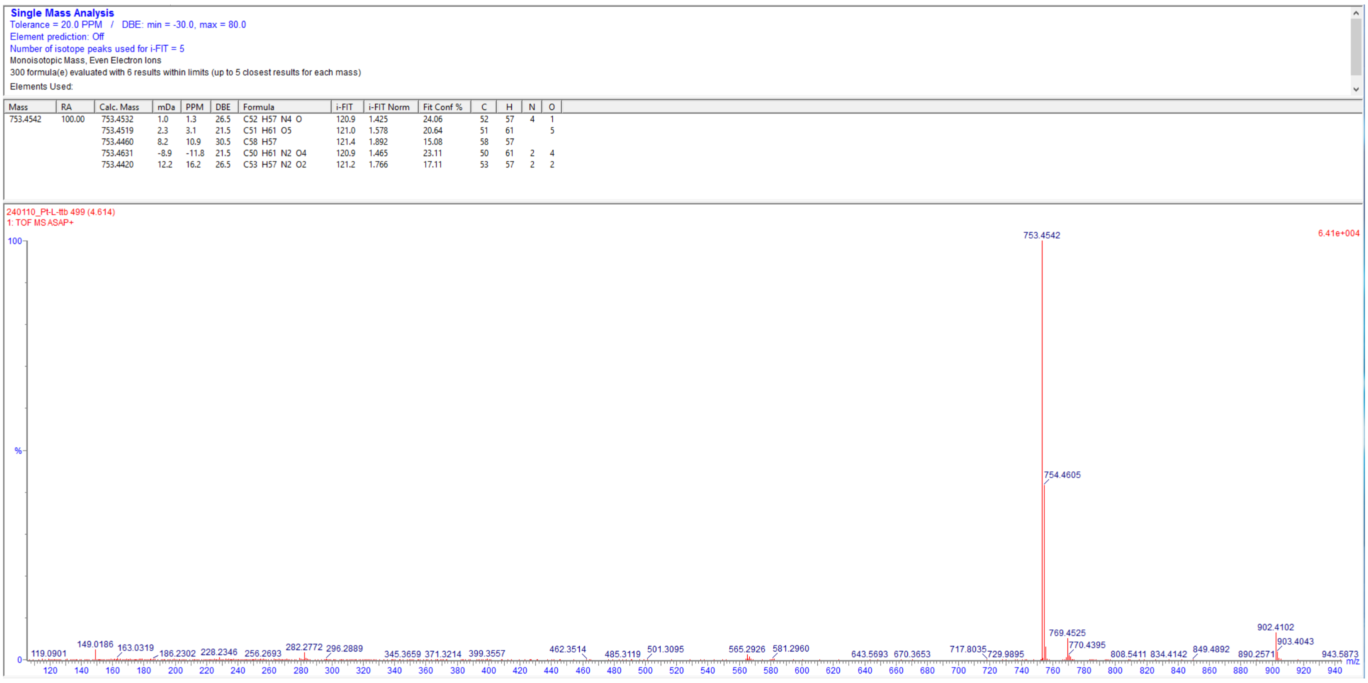

Supplementary Figure 16. QTof mass data of Intermediate material (5)

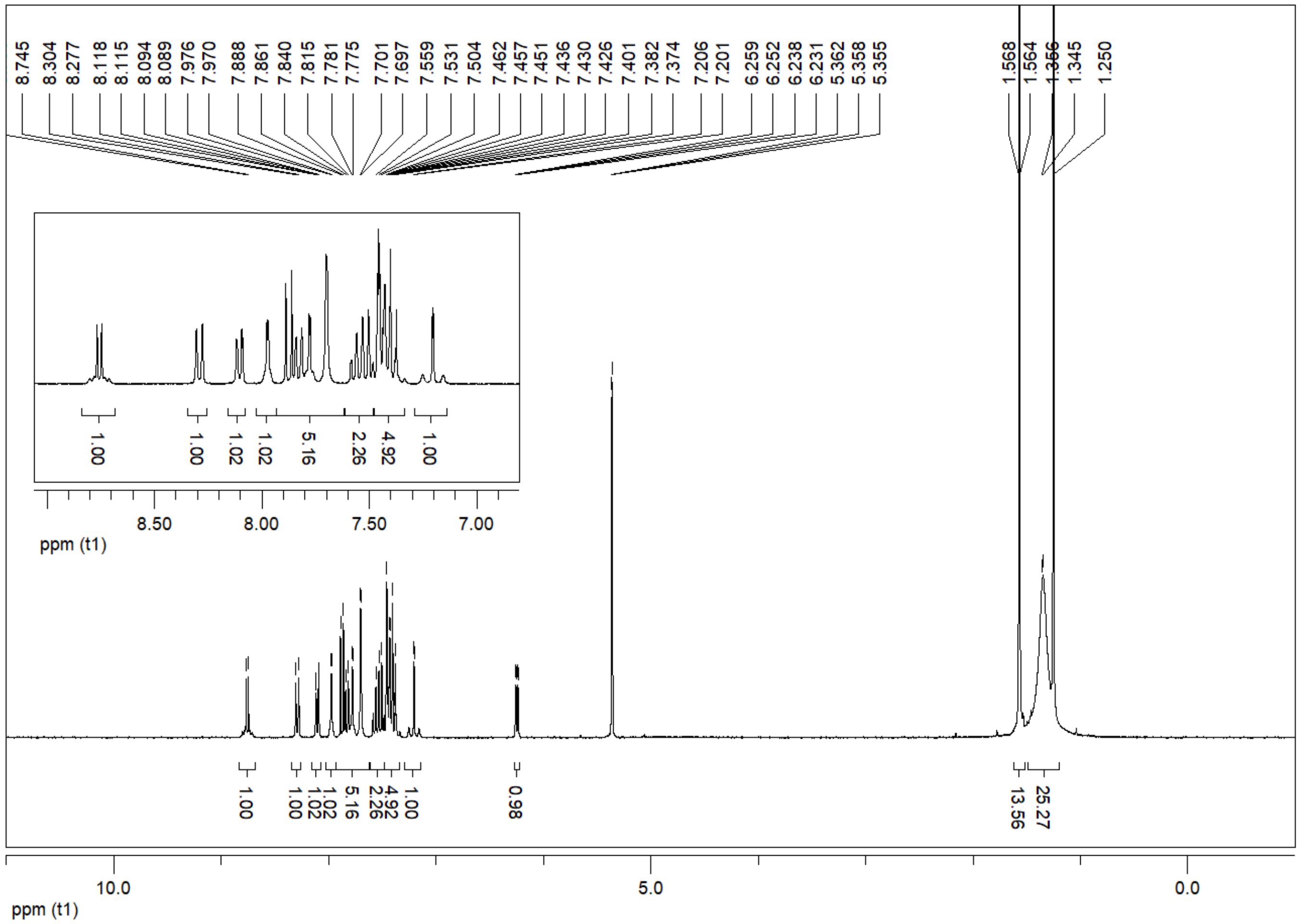

Supplementary Figure 17. ^{1}H-NMR spectrum of PtON-tb-TTB

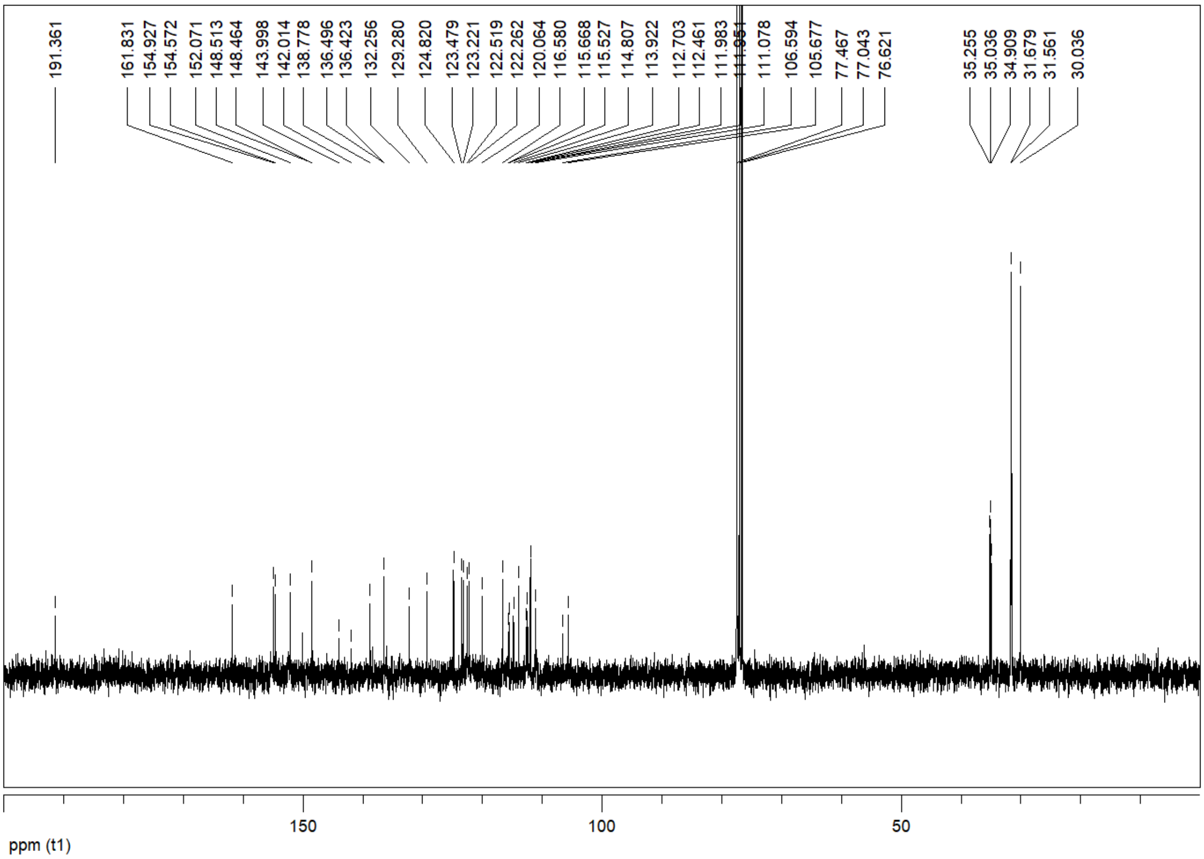

Supplementary Figure 18. ^{13}C-NMR spectrum of PtON-tb-TTB

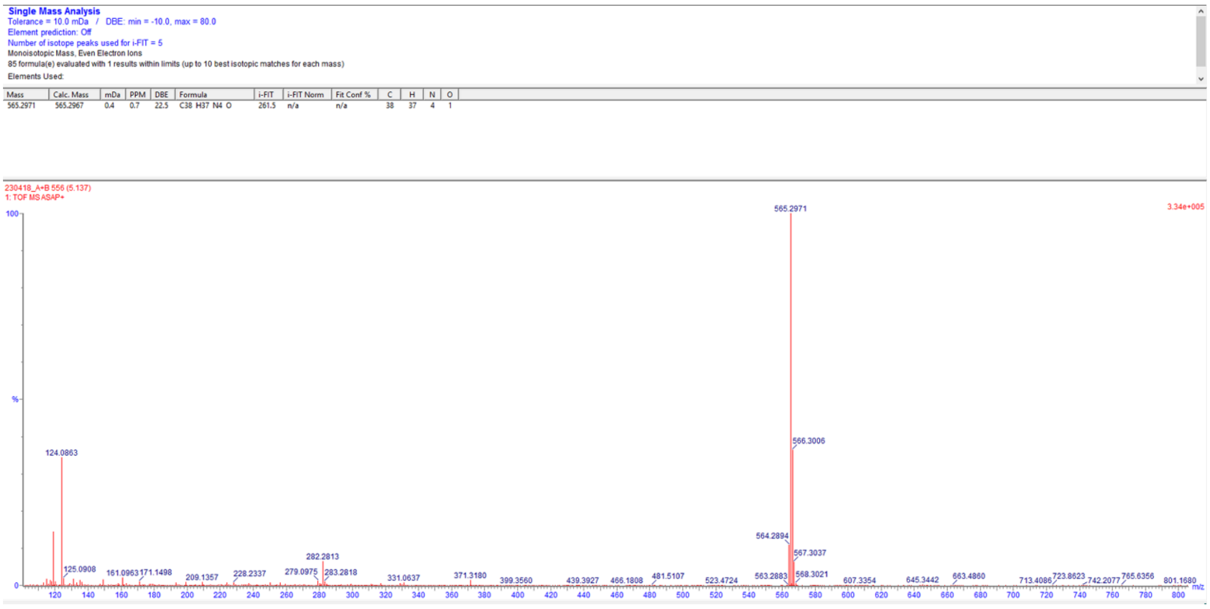

Supplementary Figure 19. QTof mass data of PtON-tb-TTB

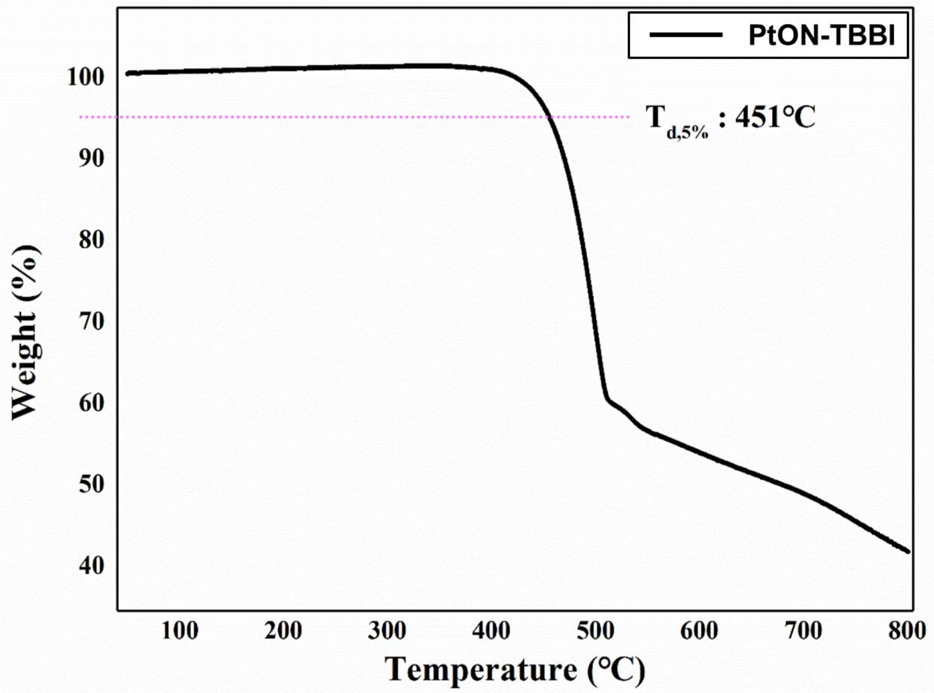
PtON-TBBI
T d,5% : 451°C
Weight (%)
Temperature (°C)

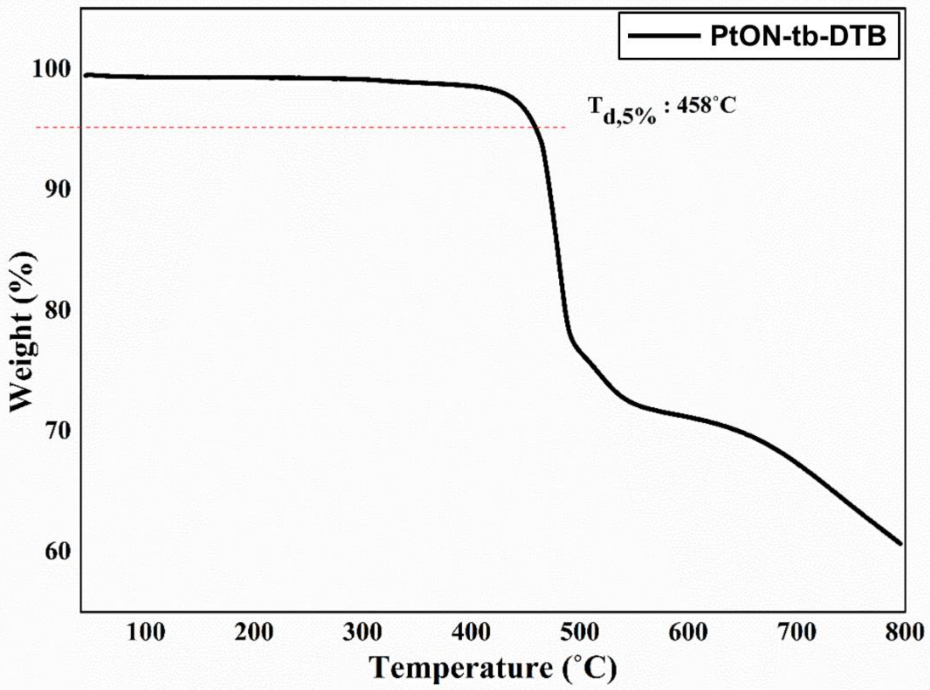
PtON-tb-DTB
T d,5% : 458°C
Weight (%)
Temperature (°C)

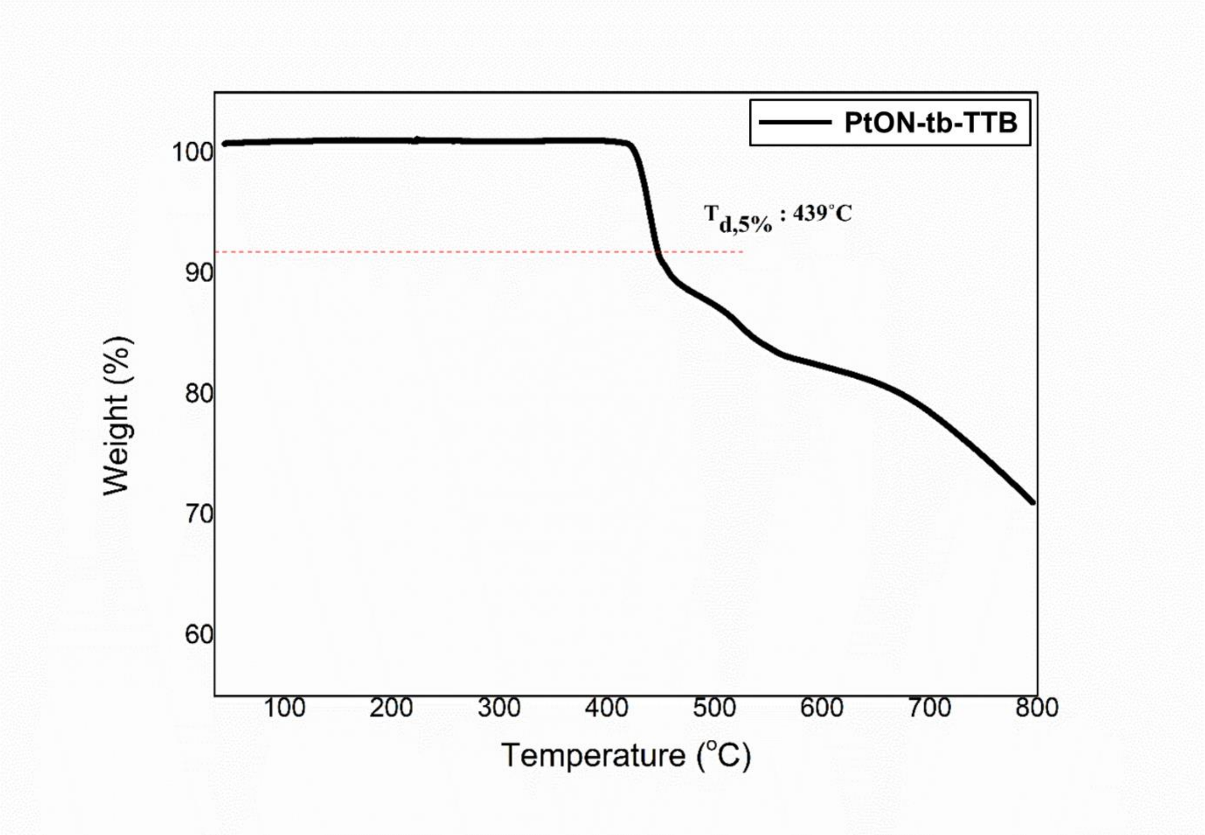

Supplementary Figure 20. TGA thermograms of PtON-TBBI, PtON-tb-DTB, and PtON-tb-TTB.

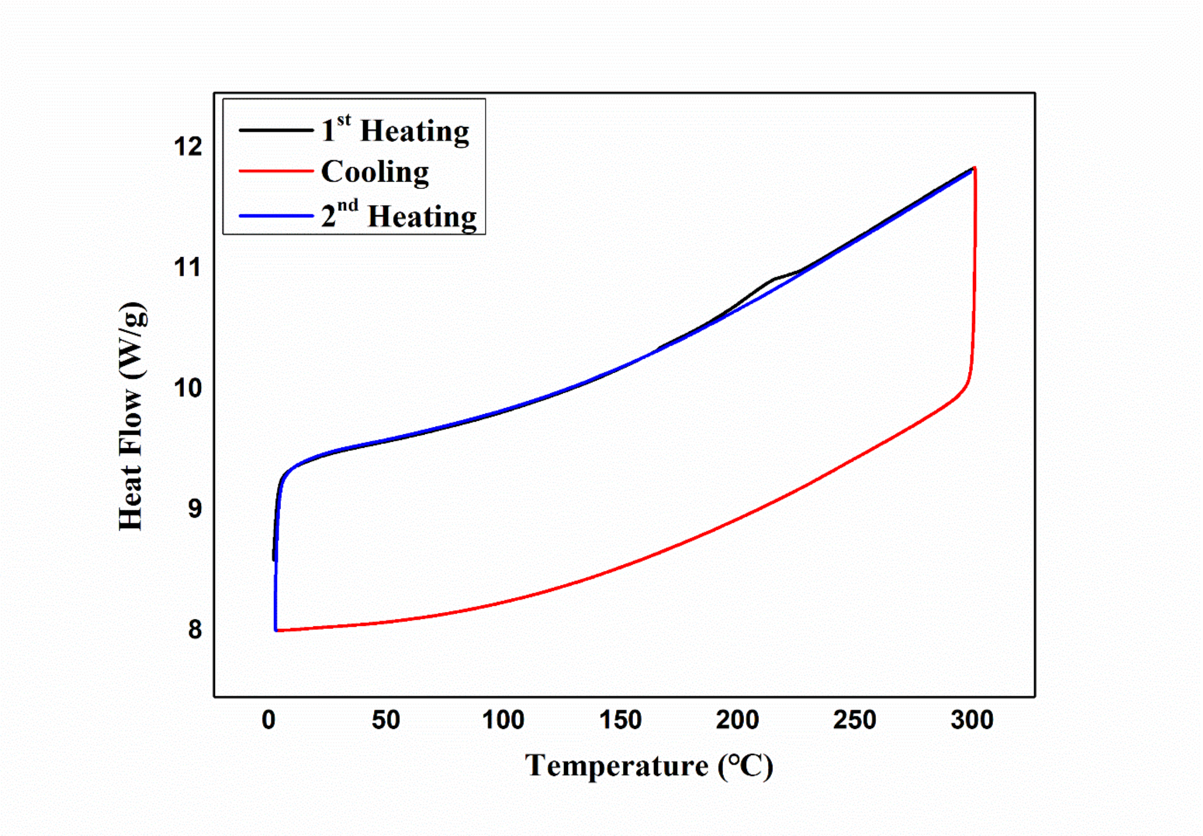

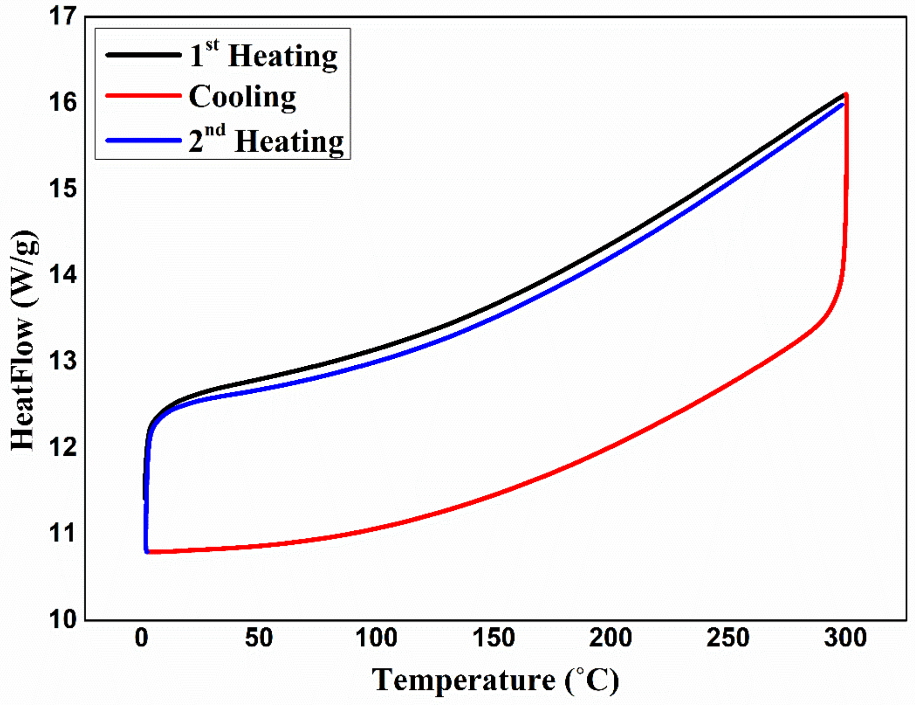

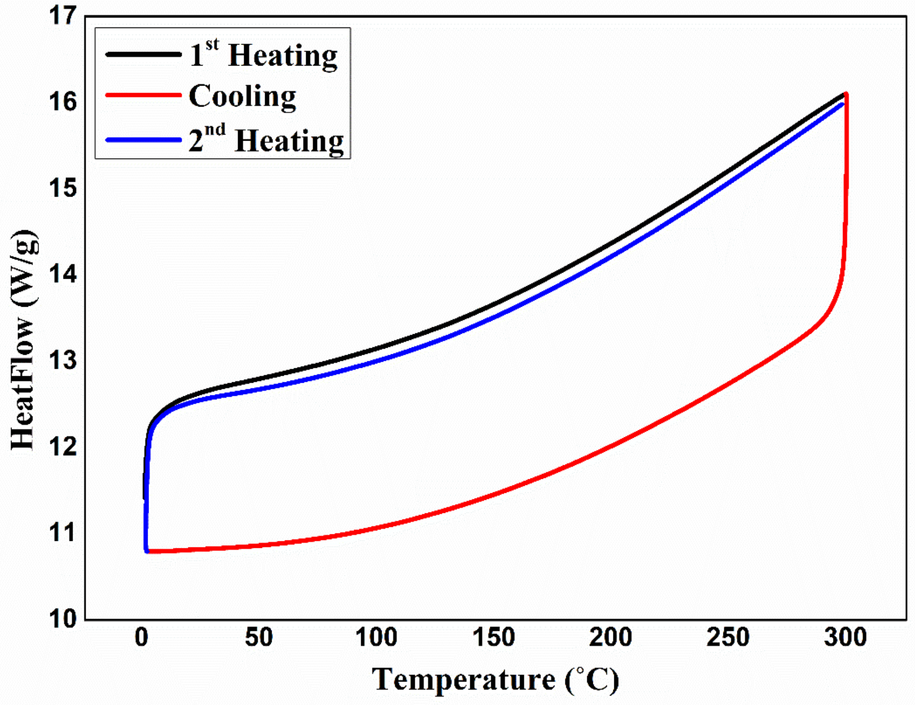

Supplementary Figure 21. DSC thermograms of platinum complexes (DSC data for PtON-TBBI, PtON-tb-DTB, Pt-tb-TTB are shown in the top figure in order).

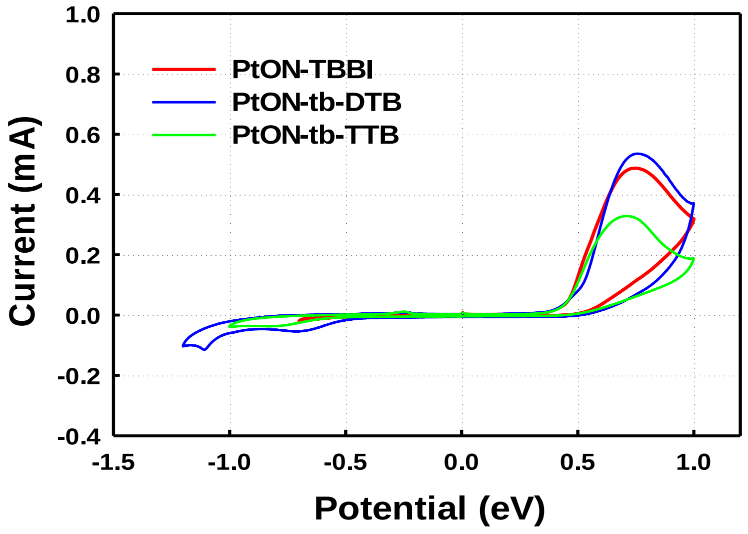

Supplementary Figure 22. Cyclic-Voltammetry measurements of PtON-TBBI, PtON-tb-DTB, Pt-tb-TTB.

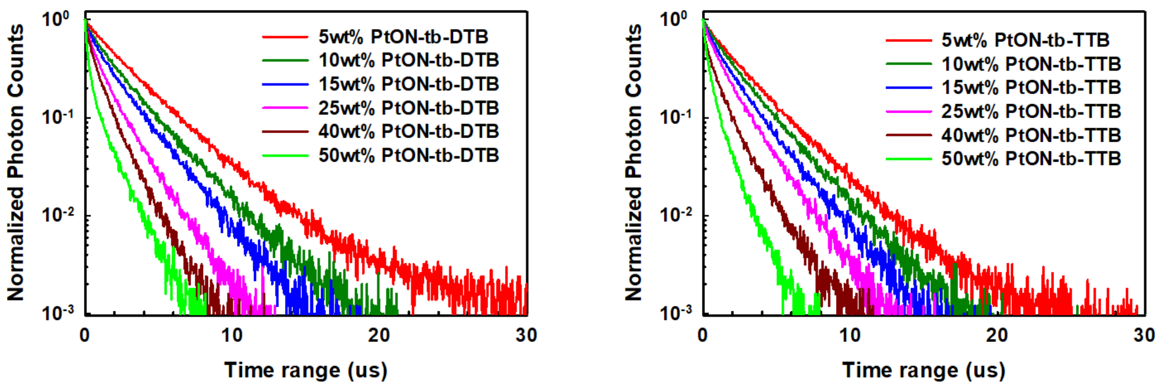

Supplementary Figure 23. TRPL measurements of PtON-tb-DTB and PtON-tb-TTB according to doping concentration on PMMA matrix.

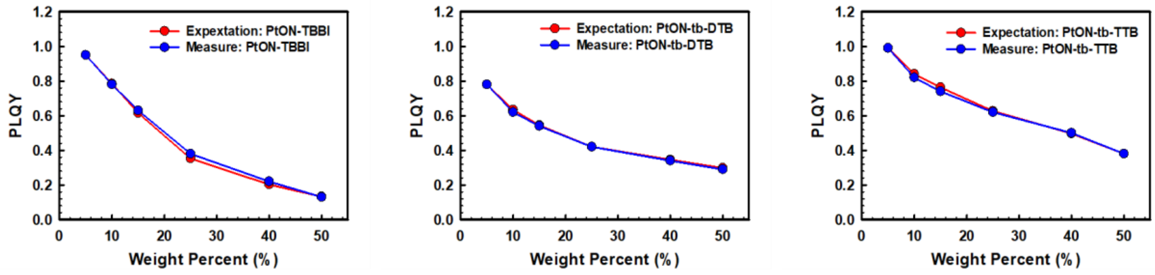

Supplementary Figure 24. Expected and Measured PLQY values of PtON-TBBI, PtON-tb-DTB, and PtON-tb-TTB according to doping concentration in PMMA matrix.

2. Device fabrication

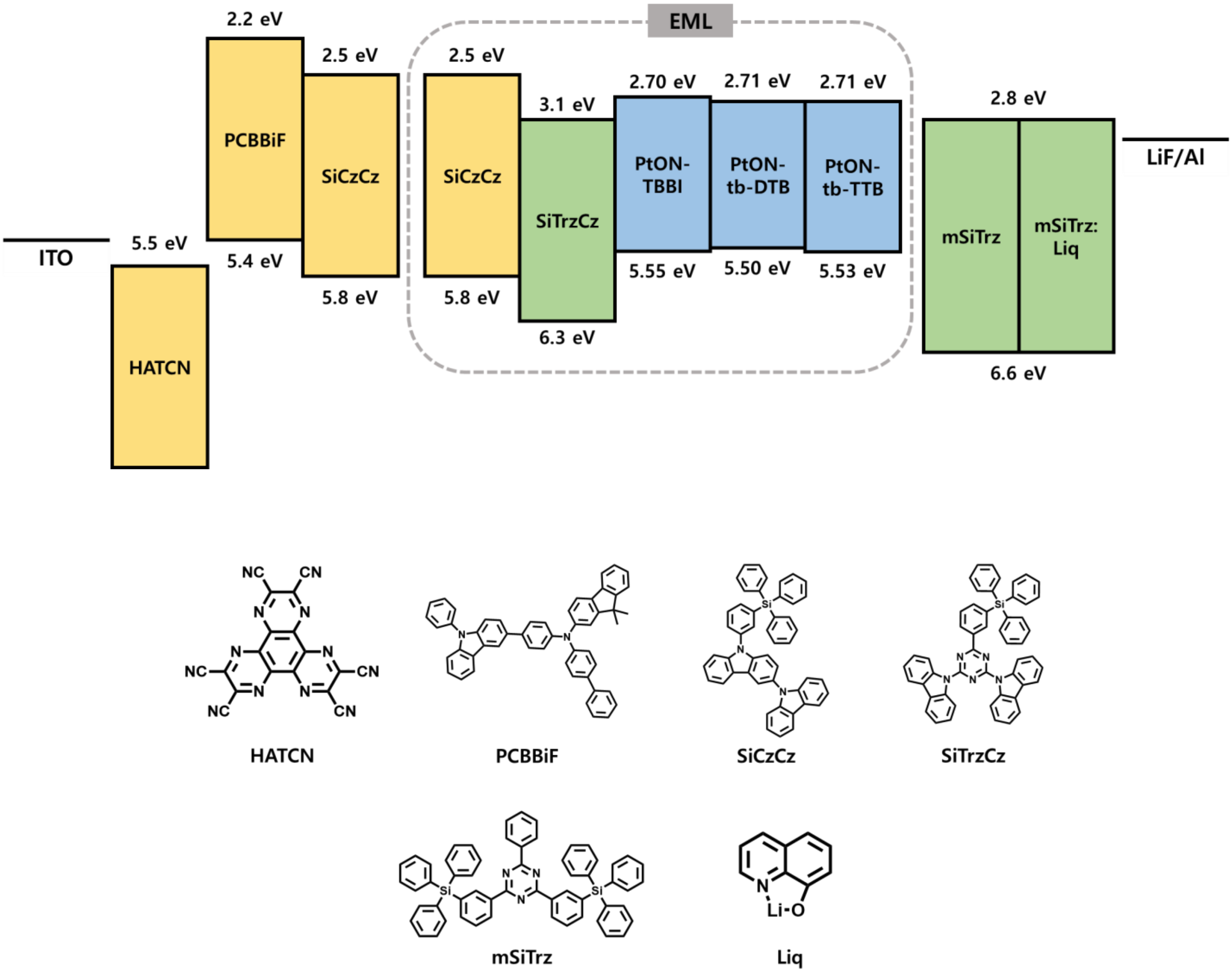

Supplementary Figure 25. Device energy diagram and molecular structures.

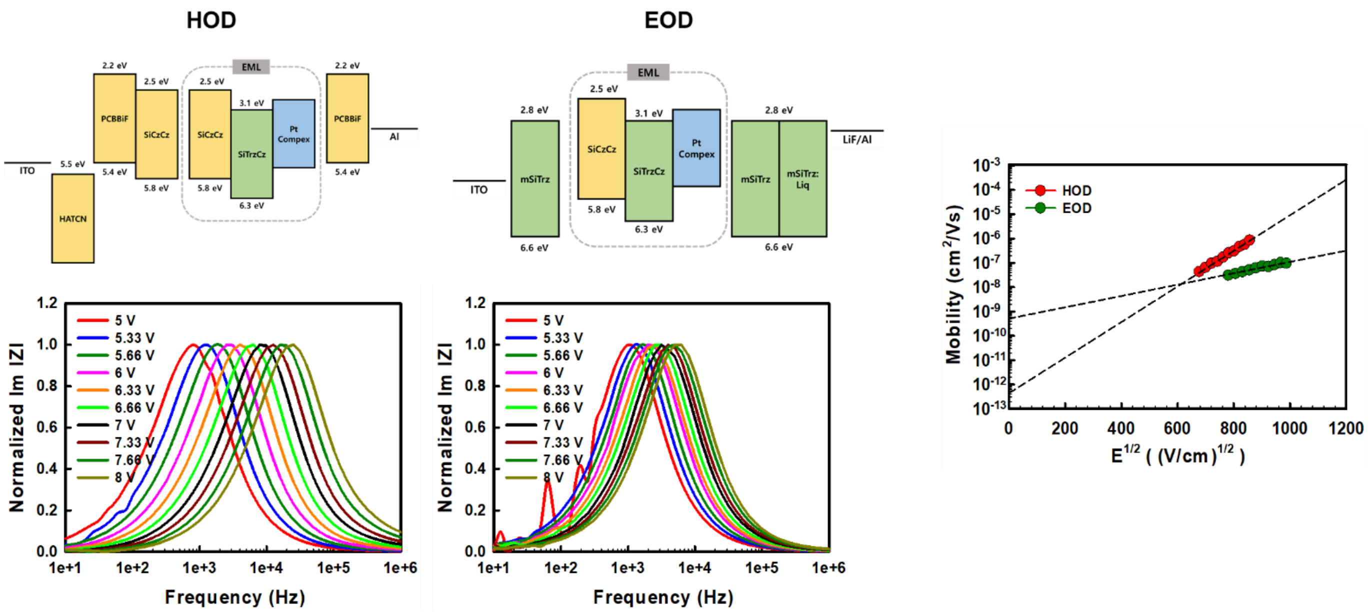

Supplementary Figure 26. Normalized Im$|Z|$ – Frequency measurements of HOD and EOD. And mobility calculation. Device configuration of HOD is ITO (50nm)/ HATCN (7nm)/ PCBBiF (45nm)/ SiCzCz (10 nm)/ 53 wt% SiCzCz: 25wt% SiTrzCz: 12wt% PtON-TBBI (40nm)/ PCBBiF (7nm), and EOD is ITO (50nm)/ mSiTrz (7 nm)/ 53 wt% SiCzCz: 25wt% SiTrzCz: 12wt% PtON-TBBI (40nm)/ mSiTrz (5nm)/ mSiTrz: Liq (2:8) (35nm)/ LiF (1.5nm)/ Al (100 nm) [2, 3].

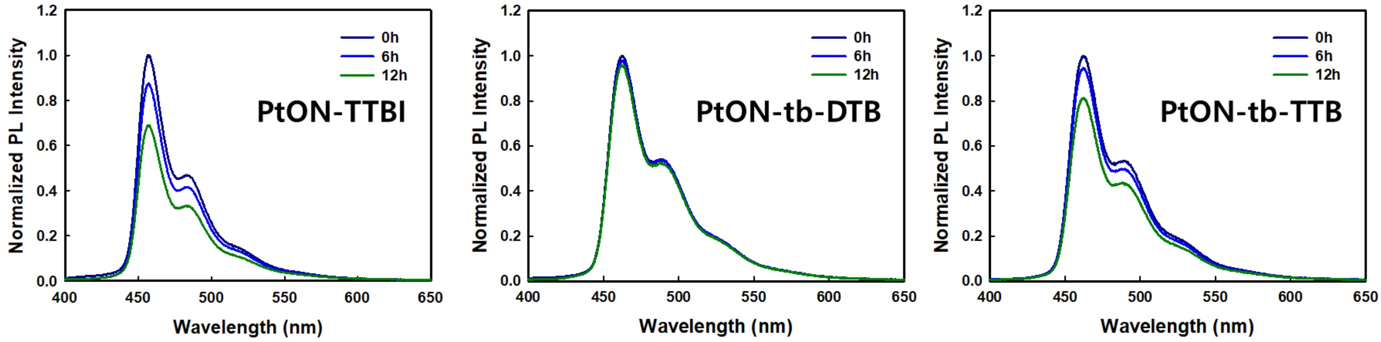

Supplementary Figure 27. UV-stability test of PtON-TBBI, PtON-tb-DTB, and PtON-tb-TTB (UV-Lamp: 360nm).

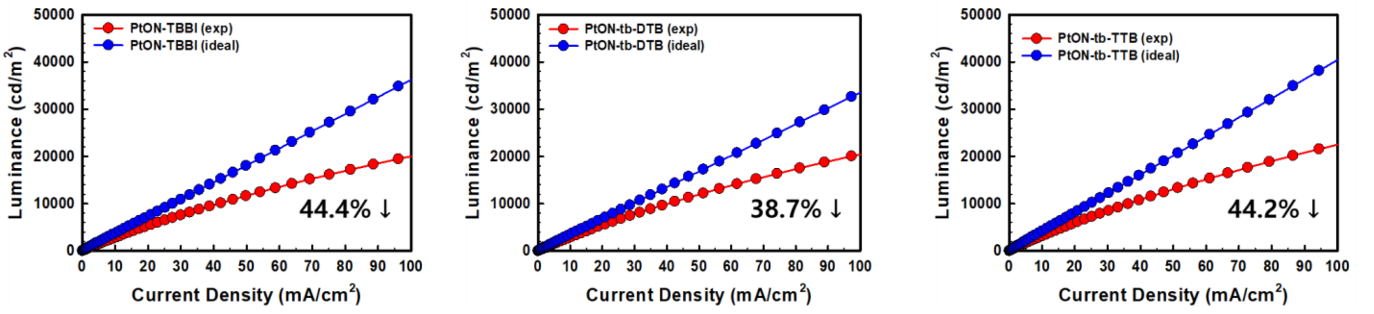

Supplementary Figure 28. Luminance (L) vs Current Density (J) graph and Luminescence decline rate (at J=100mA/cm^2) of PtON-TBBI, PtON-tb-DTB, and PtON-tb-TTB

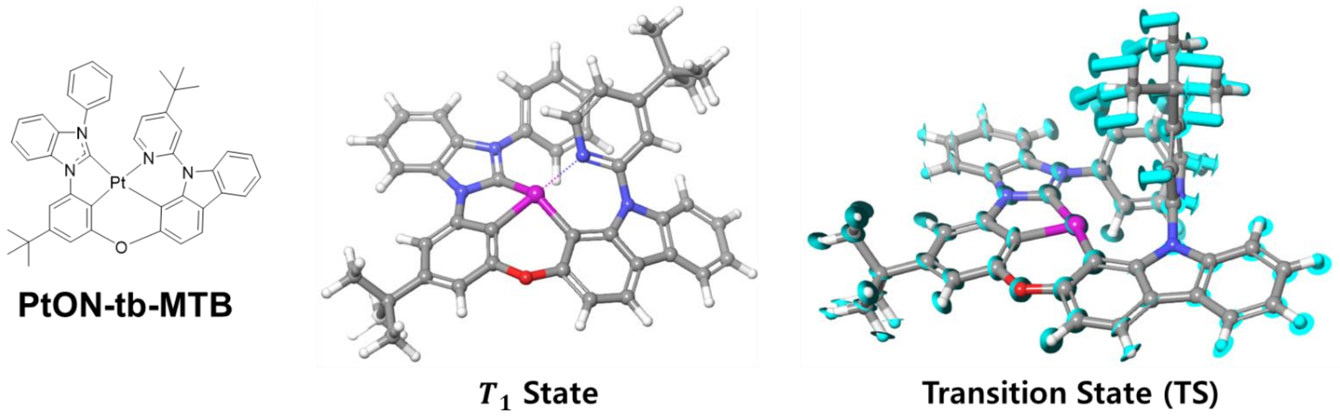

	∠C1-N1-C2-C3 (T_1)	∠C4-Pt-N2-C5 (T_1)	∠C6-N3-C5-N2 (TS)
PtON-tb-MTB	42.5 °	23.3 °	-109.6 °
PtON-tb-DTB	54.7 °	20.3 °	-90.7 °

Supplementary Figure 29. DFT simulation results of PtON-tb-MTB

3. Supplementary Tables

Supplementary Table 1. Measured exciton lifetime according to doping ratio.

	$\tau_{5wt\%}$(μs)	$\tau_{10wt\%}$(μs)	$\tau_{15wt\%}$(μs)	$\tau_{25wt\%}$(μs)	$\tau_{40wt\%}$(μs)	$\tau_{50wt\%}$(μs)
PtON-TBBI	2.64	2.18	1.71	0.98	0.56	0.37
PtON-tb-DTB	3.25	2.64	2.27	1.75	1.44	1.24
PtON-tb-TTB	2.97	2.52	2.29	1.88	1.49	1.14

Supplementary Table 2. Calculated FRET and DET rate according to intermolecular distance.

R (nm)		2.77	2.42	2.04	1.74	1.62
PtON-TBBI	k_{ET}^{F} (10^4)	0.14	0.32	0.88	2.3	3.5
	k_{ET}^{D} (10^6)	0.079	0.20	0.63	1.4	2.3

R (nm)		2.76	2.42	2.04	1.74	1.62
PtON-tb-DTB	k_{ET}^{F} (10^4)	0.065	0.15	0.41	1.0	1.6
	k_{ET}^{D} (10^6)	0.070	0.13	0.26	0.38	0.48

R (nm)		2.87	2.50	2.11	1.80	1.68
PtON-tb-TTB	k_{ET}^{F} (10^4)	0.032	0.072	0.20	0.51	0.80
	k_{ET}^{D} (10^6)	0.060	0.099	0.19	0.34	0.53

Supplementary Table 3. Calculated zero-field mobility and Poole-Frenkel constants of HOD and EOD.

$\mu_{hole,0}$ (cm^2/Vs)	$\beta_{hole,PF}$ $(cm^{\frac{1}{2}}/V^{\frac{1}{2}})$	$\mu_{electron,0}$ (cm^2/Vs)	$\beta_{hole,PF}$ $(cm^{\frac{1}{2}}/V^{\frac{1}{2}})$
4.53×10^{-13}	1.69×10^{-2}	5.13×10^{-10}	5.36×10^{-13}

4. Supplementary Discussion

4.1 Forster Resonance Energy Transfer (FRET) Rate

Forster Resonance Energy Transfer (FRET) Rate: $k_{ET}^{F} = \frac{1}{\tau_D}(\frac{R_0}{R})^6$, ${R_0}^6 = \frac{9000(ln10)\Phi_p {\kappa_p}^2}{N_A 128\pi^5 {n_D}^4} J(\lambda)$ (1)

Intermolecular distance: $R = 2 \times \sqrt[3]{\frac{3M_W}{4\pi\rho\beta N_A}}$ (2)

To calculate FRET rate, Forster Radius (R_0) should be calculated. Avogadro number (N_A), dipole orientation (${\kappa_p}^2$), and refractive index (n_D) are used as constant. Exciton lifetime (τ_D) and PLQY (Φ_p) are extracted by 5wt% doped PMMA film. as a result, k_{ET}^{F} can be obtained by calculating intermolecular distance (R).

4.2 Dexter Energy Transfer (DET) Rate

Dexter Energy Transfer (DET) Rate: $k_{ET}^{D} = KJ_T e^{-\frac{2R}{L}}$ (3)

By using measured exciton lifetime according to doping concentration, k_{ET}^{D} was calculated by using k_{ET}^{F} through equation (4) in the main scripts. Calculated DET rate is fitted by using y=A*exp(-B*x) function. Through the fitted results, KJ_T and L values are obtained.

4.3 Roll-off Analysis.

Langevin Recombination (LR) rate: $\gamma = \frac{q(\mu_n+\mu_p)}{\varepsilon\varepsilon_0}$ (4)

Roll-off model: $\frac{dP}{dt} = \frac{J}{qd} - \gamma P^2$, $\frac{dT}{dt} = -\frac{1}{\tau_D}T - \frac{1}{2}k_{TT}T^2 - k_{TP}TP + \gamma P^2$ (5)

Steady state condition is used to calculate k_{TT} and k_{TP} ($\frac{dP}{dt}$=0, $\frac{dT}{dt}$=0). Langevin Recombination (LR) rate at 10^6 V/cm is obtained from Capacitance-Voltage measurements presented on **Supplementary Figure 25**. By using LR rate and steady state condition, polaron density can be obtained according to current density. [EQE (J) $= EQE_{max} \frac{T}{T_0}$] is relation equation between EQE and triplet density. T and T_0 is the triplet density at the ($k_{TT}, k_{TP} \neq 0$) and ($k_{TT}, k_{TP} = 0$), respectively. By using J_0, relation equation between k_{TT} and k_{TP} can be obtained. Through utilization of above-mentioned relation equations, EQE-J curve can be fitted.

5. Supplementary References

1. Sun, J., Ahn, D., Kang, S., Ko, S. B., Song, D., Um, H. A., Kim, S., Lee, Y., Jeon, P., Hwang, S. H., You, Y., Chu, C. & Kim, S. Exceptionally stable blue phosphorescent organic light-emitting diodes, *Nat. Photonics*, **16**, 212-218 (2022).

2. Kim. J. M. & Kim, J.J. Charge transport layers manage mobility and carrier density balance in light-emitting layers influencing the operational stability of organic light emitting diodes, *Org. Electron.*, **67**, 43-49 (2019).

3. Kim. J. M., Lee, C. H., & Kim, J.J. Mobility balance in the light-emitting layer governs the polaron accumulation and operational stability of organic light-emitting diodes, *Appl. Phys. Lett.*, **111**, 203301 (2017).
